# Contrasting patterns of molecular evolution in metazoan germ line genes

**DOI:** 10.1186/s12862-019-1363-x

**Published:** 2019-02-11

**Authors:** Carrie A. Whittle, Cassandra G. Extavour

**Affiliations:** 1 Department of Organismic and Evolutionary Biology, Harvard University, 16 Divinity Avenue, Cambridge, MA 02138 USA; 2 Department of Molecular and Cellular Biology, Harvard University, 16 Divinity Avenue, Cambridge, MA 02138 USA

**Keywords:** Germ line genes, Primordial germ cells, Protein divergence, Codon usage, Molecular evolution, Metazoans, Specification mode, *Drosophila*, *Caenorhabditis*, *Apis*

## Abstract

**Background:**

Germ lines are the cell lineages that give rise to the sperm and eggs in animals. The germ lines first arise from primordial germ cells (PGCs) during embryogenesis: these form from either a presumed derived mode of preformed germ plasm (inheritance) or from an ancestral mechanism of inductive cell-cell signalling (induction). Numerous genes involved in germ line specification and development have been identified and functionally studied. However, little is known about the molecular evolutionary dynamics of germ line genes in metazoan model systems.

**Results:**

Here, we studied the molecular evolution of germ line genes within three metazoan model systems. These include the genus *Drosophila* (*N*=34 genes, inheritance), the fellow insect *Apis* (*N*=30, induction), and their more distant relative *Caenorhabditis* (*N*=23, inheritance). Using multiple species and established phylogenies in each genus, we report that germ line genes exhibited marked variation in the constraint on protein sequence divergence (dN/dS) and codon usage bias (CUB) within each genus. Importantly, we found that *de novo* lineage-specific inheritance (LSI) genes in *Drosophila* (*osk*, *pgc*) and in *Caenorhabditis* (*pie-1*, *pgl-1*), which are essential to germ plasm functions under the derived inheritance mode, displayed rapid protein sequence divergence relative to the other germ line genes within each respective genus. We show this may reflect the evolution of specialized germ plasm functions and/or low pleiotropy of LSI genes, features not shared with other germ line genes. In addition, we observed that the relative ranking of dN/dS and of CUB between genera were each more strongly correlated between *Drosophila* and *Caenorhabditis*, from different phyla, than between *Drosophila* and its insect relative *Apis*, suggesting taxonomic differences in how germ line genes have evolved.

**Conclusions:**

Taken together, the present results advance our understanding of the evolution of animal germ line genes within three well-known metazoan models. Further, the findings provide insights to the molecular evolution of germ line genes with respect to LSI status, pleiotropy, adaptive evolution as well as PGC-specification mode.

**Electronic supplementary material:**

The online version of this article (10.1186/s12862-019-1363-x) contains supplementary material, which is available to authorized users.

## Background

Germ lines are the specialized cell lineage contained in the gonads of sexually reproducing animals that give rise to the sperm and eggs. The germ cell lineages are separate from the soma and are the only source of heritable genetic variation passed between generations, providing them with a crucial role in reproductive success, fitness and evolutionary biology. Extensive experimental and cytological research has focused on the discovery and functionality of germ line genes in animal models, which has led to the identification of a wide range of genes involved in germ line establishment and development [1–5]. At present however, much remains unknown about the molecular evolutionary dynamics of these germ line genes within metazoan model systems.

The germ lines first emerge in early embryogenesis with the formation of primordial germ cells (PGCs). The PGCs in some organisms arise from a presumed evolutionarily derived mode of maternally generated germ line determinants (inheritance), otherwise known as germ plasm (specialized cytoplasm containing proteins and RNAs), as has been observed in systems including flies (*Drosophila*), wasps (*Nasonia*), nematodes (*Caenorhabditis*), and frogs (*Xenopus*) [2, 4, 6–9]. Alternatively, PGCs may emerge from an ancestral mechanism involving inductive cell-cell signalling pathways (induction) as has been supported by experimental embryological and functional genetic data in taxa such as crickets (*Gryllus*), mice (*Mus*), and salamanders (*Ambystoma*) [4, 5, 8–12]. Gene products involved in germ line specification and in various stages of germ line development have been identified using different model systems (*e.g.,* [1–5, 13–16]), allowing study of how those genes involved in core germ line functions have evolved.

A primary model for the study of germ line genes has been *Drosophila*. In *Drosophila*, PGC-specification depends on maternally provided germ plasm (inheritance), which is asymmetrically located in the posterior region of the oocyte cytoplasm [4, 17]. Genes essential to germ plasm formation and PGC specification in *Drosophila* include two genes believed to have originated only in insects, *oskar* (or *osk*) and *pgc* (*polar granule component*) [2, 4, 18, 19]. *osk* is involved in the recruitment of most other germ plasm components in *D. melanogaster* [2, 4]. *pgc* is involved in transcriptional repression in pole cells, and may be exclusive to *Drosophila*, as this gene has not been reported in other Dipteran insects or other animals to date [19, 20]. Together, these two genes, *osk* and *pgc* appear to be *de novo* genes [21] originating in specific insect lineages that may have facilitated the evolutionary transition to germ plasm and evolved crucial functions in early stages of germ line development, that is PGC specification [2, 4, 5, 17, 19].

Another central model for germ line gene research is the nematode *C. elegans*. In this taxon, similar to *Drosophila*, PGCs are specified under the inheritance mode, via localized molecular entities denoted in that system as P granules [3, 22]. Rather than presence alone, a sufficient concentration and specific conformation of P granules are required for them to act as germ line determinants [23–25]. P granules contain protein products of nematode-specific genes, including the RNA-binding *pgl-1 (P-granule abnormality*) (a partly redundant *pgl-3* also plays a role; [26]), which helps keep P granule components localized and thus functional [4, 26]. *meg-1* (*maternal-effect germ cell defective*), which has been linked to P-granule assembly and fertility [*meg-2* has also been identified as putatively partly redundant; [27, 28]), may be specific to the species *C. elegans*, as it appears to display poor homology to any other animal proteins, including within the same genus [3, 27]. In addition, the *Caenorhabditis* gene *pie-1* (*pharyngeal and intestinal excess*), is involved in transcriptional repression and maintaining germ cell fate [29], and shares functional (but not sequence) similarity to *D. melanogaster pgc* [1, 4, 19]. This gene is essential for PGC establishment and has only been reported in nematodes [4]. In this regard, whilst the inheritance mode (germ plasm) is shared between *Drosophila* and *Caenorhabditis*, the key upstream regulators appear to have arisen independently and in a lineage-restricted manner in these two model organisms [2–4, 19, 30], consistent with the proposed convergent evolution of this mechanism [7, 9].

An additional primary model of study of germ line genes including those involved in PGC-specification, this time using induction mode, or zygotic cell-cell signalling, is mouse [4, 5, 17]. Whilst much of the genetic programming remains to be understood, it has been established that in early embryogenesis ligands including BMPs (Bone Morphogenetic Proteins; particularly BMP2 and BMP4) and WNT3 are crucial for inducing signals that initiate PGC formation [4, 5]. Such signalling activates expression of the transcription factor *Blimp-1* (*B lymphocyte-induced maturation protein-1*), needed for repression of somatic gene expression, epigenetic programming of the genome, and ultimately PGC formation [4, 5, 17, 31]. Recently, similar processes involving *BMP* and *Blimp-1* signalling have been shown to be utilized in the induction of PGCs in a basally branching insect, the cricket *Gryllus bimaculatus* [10, 32] suggesting conservation of this signalling mechanism across these divergent taxa with the induction mode. Although the precise nature of the signalling pathways that may induce PGCs in other insects remains largely unknown, unlike in *Drosophila*, in some insects PGCs are first described as arising from among the mesoderm of the abdominal segments in the vicinity of the future primordial gonad [33]. This is the case, for example, in the honeybee *Apis mellifera*, where cells with the morphology and gene expression typical of animal PGCs are first detected late in embryogenesis in the abdominal segments that will house the primordial gonad [34–38]. Thus, we infer that BMP/Blimp-1 signalling to induce the germ line may likely be shared with other insect models that appear to specify PGCs via inductive-signalling [33] such as *Apis*.

Many germ line genes, including specification genes, appear to play multiple roles in germ line development and/or across animal models [1, 3, 4, 13–16]. The gene *vasa*, an ATP-dependent RNA helicase, is a universal marker of germ lines [1, 39] (although in some animals it plays somatic roles (reviewed by [40]). The products of *vasa* are essential components of *Drosophila* germ plasm [18, 41, 42], localize to *C. elegans* P granules (the *vasa* ortholog *glh-1* is essential for fertility; *glh-2-4* may be nonessential; [43, 44]), and become upregulated in mouse and cricket PGCs following their induction (*vasa* mouse ortholog is *mvh*: 10, 32, [45, 46]), consistent with a ubiquitous role in germ lines across these divergent systems. In addition, *nanos* (or *nos*) and *pumilio* (*pum*) encode interacting RNA-binding proteins which have each been linked to germ plasm and PGCs in *Drosophila* and been associated with early germ line development across metazoans (reviewed by [1, 4, 47]). In sum, current data suggest that while certain germ line genes are particularly involved in either the inheritance and induction modes [1–5], many germ line genes play various roles across modes and/or throughout germ line development [1, 3, 4, 13–16].

At present, investigations into the molecular evolution of germ line genes remain uncommon, and the few studies available have largely focused on putative genetic regulators in the germ line stem cells of *Drosophila*. For instance, a recent study in *Drosophila* examined 366 genes identified from RNAi screening as involved in adult self-renewing female germ line stem cells [48]. That study suggested those genes were increased targets of short-term selective sweeps, but not typically of recurrent long-term (interspecies) positive selection based on dN/dS [48]. Separate assessments have reported that certain genes expressed in adult germ lines including stem cells (e.g. *pum*, *stone-wall* (*stwl*)) have evolved adaptively and/or in response to Wolbachia infection in *Drosophila* [49–51]. In terms of the investigation of germ line genes involved in PGC specification [19, 52], a study of the *osk* gene in *Drosophila* has shown that dN/dS varies among segments of this protein and shows some signs of positive selection. However, these signs were reported for only one of the 18 species studied (*D. virilis*), and the signal was not consistent across various approaches even in that lineage [52]. Given the limited data on the molecular evolution of germ line genes further study is warranted in metazoan models.

In the present study, we investigate the molecular evolution of genes with experimental or cytological evidence of involvement in germ line specification and/or development, broadly referred to herein as germ line genes, in metazoan models. Specifically, we examine 34 genes in our main target and reference genus *Drosophila* (Phylum Arthropoda, Order Diptera), as well as 23 genes in the divergent genus *Caenorhabditis* (Phylum Nematoda, Order Rhabditida). These genera represent two cases of independently evolved inheritance mode of PGC-specification, and each contains lineage-specific inheritance (LSI) genes, essential to germ plasm functionality (*osk*, *pgc* for *Drosophila* and *pie-1* and *pgl-1* for *Caenorhabditis*). We also study 30 genes in a second insect genus, *Apis* (Phylum Arthropoda, Order Hymenoptera), a taxon likely to use the induction mode. Collectively, the results provide insights into the molecular evolutionary dynamics of germ line genes within each of three distinct metazoan model genera. Most notably, the data show that germ line genes exhibit wide variation in constraint on protein sequence evolution and codon usage within each genus. Further, the LSI genes, which are essential to germ line function and found only in certain lineages, consistently exhibit a striking history of rapid protein sequence evolution relative to other germ line genes in each respective genus. We show this pattern may be explained by adaptive evolution and/or low pleiotropy.

## Results and Discussion

We assessed the molecular evolution of 34 germ line genes across six species of the *melanogaster* group in our main target and reference genus *Drosophila* (*D melanogaster*, *D. erecta*, *D. sechellia*, *D. simulans*, *D. yakuba*, and an outgroup species *D. ananassae* (Additional file 1: Table S1 and Figure S1). The gene list included 34 genes with known experimental or cytological evidence of functionality in early germ line development, including PGC specification, and is provided in Table 1. For our study, the genes for *Drosophila* were grouped into four categories as follows: 1) the LSI genes *osk* and *pgc*, which are directly involved in germ plasm function, and found only in certain insects, including *Drosophila* [2, 19]; 2) genes involved in regulating PGC-specification under the inheritance mode (“Inheritance”, *N*=13) in *Drosophila* (and other organisms) studied to date; 3) orthologs to genes found to be involved in inductive signalling mode in mice or other models (“Induction”, *N*=15); and 4) genes involved in germ line formation regardless of mode (“Inh/Ind”, *N*=4) (Table 1). In addition to studying these 34 germ line genes in *Drosophila*, we examined identifiable orthologs (see Methods) to this gene set in *Caenorhabditis* (for categories 2-4; four species studied, Tables S1 and S2), as well as two *Caenorhabditis* LSI genes (*pie-1* and *pgl-1*; *N*=23 genes total, Additional file 1: Table S2, see Methods), and orthologs found in the fellow insect genus *Apis* (N=30 genes; four species studied; Additional file 1: Table S3), which likely exhibits induction.

**Table 1:** The 34 germ line genes studied in *Drosophila*. For this study, genes have been classified based on known roles in PGC-specification in established models. However, many genes (excluding LSI genes) have been reported to play roles in both germ line specification and at some stage(s) of germ line development or maintenance across model systems and specification modes [1, 3, 4, 13–16].

Gene	Gene Name	FlyBase ID	Length (codons)	Established PGC or Germ Line Role in Animal Models	References
Category 1: Lineage-Specific Inheritance (LSI) (*N*=2)					
*osk*	*oskar*	FBgn0003015	607	Assembly of germ plasm components	[2, 4, 18]
*pgc*	*polar granule component*	FBgn0016053	72	Localized to germ plasm; transcriptional repression in pole cells (DM)	[19, 20]
Category 2: Inheritance (*N*=13)					
*armi*	*armitage*	FBgn0041164	1189	*osk* functionality and mRNA translocation (DM)	[125]
*bru-1*	*bruno 1*	FBgn0000114	811	*osk* regulation: binding mRNA, translocation (DM)	[126]
*capu*	*cappuccino*	FBgn0000256	1362	Interacts with *spir*; Localization of *osk* mRNA, VASA and STAUFF to germ plasm, oocyte polarity (DM)	[127, 128]
*cup*	*cup*	FBgn0000392	1118	*Osk* regulation, with *bruno* (DM)	[129]
*cycB*	*cyclin B*	FBgn0000405	531	Cell cycle; mRNA localized to germ plasm (DM)	[20, 130]
*gcl*	*germ cell less*	FBgn0005695	570	Localized to germ plasm; required for PGC specification (DM)	[20]
*mago*	*mago nashi*	FBgn0002736	148	Required for PGC specification; localization of *osk* mRNA and STAUF to posterior pole of oocyte (DM)	[131]
*orb*	*oo18 RNA-binding protein*	FBgn0004882	916	Localized to germ plasm; *osk* regulation, oocyte polarity (DM)	[20, 132]
*psq*	*pipsqueak*	FBgn0263102	1124	Required for germ plasm formation	[133]
*spir*	*spire*	FBgn0003475	1021	Products are component of germ plasm (DM), interacts with *capu*	[127, 128]
*stau*	*staufen*	FBgn0003520	1027	Localization to germ plasm, PGC specification	[1, 127, 134]
*tud*	*tudor*	FBgn0003891	2516	Component of germ plasm, germ cell formation (DM)	[1, 135]
*vls*	*valois*	FBgn0003978	368	Component of germ plasm, involved in assembly (DM)	[136]
Category 3: Induction (*N*=15)					
*Blimp-1*	*Blimp-1*	FBgn0035625	1217	BMP signalling pathway for PGC specification, represses somatic expression (MM, GB)	[4, 5, 10, 17, 31, 137, 138]
*bnl*	*branchless*	FBgn0014135	771	FGF protein; mitogen for PGCs (MM)	[139, 140]
*btl*	*breathless*	FBgn0005592	1053	FGFR (FGF receptor), linked to PGCs (MM)	[140]
*byn*	*brachyury*	FBgn0011723	698	Activation of *BLIMP-1;* essential for PGC specification (MM)	[141]
*dpp*	*decapentaplegic (BMP 2/4-ortholog)*	FBgn0000490	589	BMP2/4 ortholog; involved in BMP-BLIMP1 signalling for PGC specification (MM, GB)	[5, 10, 32, 137]
*gbb*	*glass bottom boat*	FBgn0024234	456	BMP5/7/8 ortholog; involved in PGC specification (MM, GB)	[5, 32, 142]
*mad*	*mothers against dpp*	FBgn0011648	526	Ortholog to MM *smad* and GB *mad* genes*;* PGC specification (MM,GB)	[5, 142, 143]
*med*	*medea*	FBgn0011655	772	Ortholog to MM *smad4;* PGC induction (MM,GB)	[5, 142]
*punt*	*punt*	FBgn0003169	521	BMP receptor; putative ortholog in MM Bmpr2 (note; or Acvr2); associated with PGCs in GB	[32, 142, 144]; orthology match in FlyBase.org
*sax*	*saxophone*	FBgn0003317	583	BMP receptor; putative mammalian ortholog Bmpr1a/b (note: or Acvr) involved PGC specification in birds	[142]; orthology match in FlyBase.org
*smox*	*smad on X*	FBgn0025800	487	Orthologs are *smad2/3* in MM, which are transcriptional regulators needed for inductive PGC specification	[5]; orthology from FlyBase.org
*sog*	*short gastrulation*	FBgn0003463	1039	MM ortholog CHRD involved in BMP/CHRD signalling pathway, which relates to PGCs; Regulates DPP (DM)	[145, 146]; Orthology from FlyBase.org
*tkv*	*thick veins*	FBgn0003716	576	BMP receptor; mammalian ortholog (Bmpr1a/b) involved PGC specification in birds, GB	[142]; orthology match in FlyBase.org
*wg*	*wingless*	FBgn0284084	469	Ortholog to MM *wnt genes*; essential for PGC specification	[5, 147]
*wit*	*wishful thinking*	FBgn0024179	914	BMP receptor; putative ortholog to MM Bmpr2 (similar to punt), GB-punt is linked to PGCs in GB	[32, 144]; orthology match in FlyBase.org
Category 4: Inh/Ind (*N*=4)					
*nos*	*nanos*	FBgn0002962	402	Germ plasm component (DM), regulates mRNA, associated with PGCs in MM; common germ cell across metazoans	[1, 4, 17, 20, 148]
*piwi*	*P-element induced wimpy testis*	FBgn0004872	844	Component of germ plasm (DM); germ line essential across metazoans (DM, MM)	[1, 149]
*pum*	*pumilio*	FBgn0003165	1534	Regulate mRNA in PGCs (DM), conserved germ cell role in humans	[1, 150]
*vasa*	*vasa*	FBgn0283442	662	mRNA and protein localizes to germ plasm (DM, zebrafish, *C. elegans*), PGCS in Xenopus and MM	[1, 149, 151, 152]

NOTE: Gene identifiers are from the reference species *D. melanogaster*. Genes were placed in one of four categories based on their role in PGC-specification. Experimental or cytological evidence linking genes to lineage-specific inheritance (LSI), to Inheritance or Induction modes, or Inh/Ind are shown. This gene list was used as a reference to identify orthologs in five other *Drosophila* species from the melanogaster group, and in *C. elegans* and *A. mellifera* using reciprocal BLASTX. Length is for the full CDS per gene. Species abbreviations in cited evidence are *Drosophila melanogaster* (DM), *Gryllus bimaculatus* (GB), or *Mus musculus* (MM).

Molecular evolution in each genus was analyzed fully independently (using within-genus alignments), allowing us to evaluate how these genes evolve in each taxon (cf. [53, 54]). We determined dN/dS, dN and dS for each phylogeny using the free-ratio model (M1) in PAML [55], a model which allows all species branches to have independent values, and using the one-ratio model (M0) which estimates a phylogeny-wide value for each parameter [55–57]. Typically, dN/dS values >1, =1 and <1 indicate positive selection, neutral evolution and purifying selection, respectively [55]. However, even when <1, elevated values of dN/dS are indicative of greater rates of protein sequence evolution and reduced constraint. Thus, dN/dS provides a means to assess the relative constraint among genes involved in specific developmental processes [58]. The distributions of dN/dS, dN and dS for all genes studied in each of the three genera are shown in box plots in Additional file 1: Figure S2 and S3 (values for free-ratio model per branch are shown, see also Tables S4-S6). As indicated therein, dN and dS were unsaturated for all species branches in *Drosophila*, *Caenorhabditis* and *Apis* with values <<1 (Additional file 1: Figure S3A-F). The only species branch among all genera nearing saturation for dS was in the outgroup of *Drosophila*, *D. ananassae*, which had a median value of 1.158, and 25^th^ and 75^th^ percentiles values of 0.989 and 1.705, remaining in a suitable range for analysis [53, 59]. To study dN/dS of each gene per genus, we determined Mean dN/ Mean dS ($\overline{\mathrm{dN}}$/$\overline{\mathrm{dS}}$) across all terminal species branches per phylogeny from the free-ratio model. Values obtained using this approach were strongly correlated to the model M0 dN/dS values within each genus (Spearman’s ranked correlation R=0.95, 0.99 and 0.99 for genes studied in *Drosophila*, *Caenorhabditis* and in *Apis* respectively, *P*<2X10^-7^). We present results for $\overline{\mathrm{dN}}$/$\overline{\mathrm{dS}}$ throughout, as this provides a phylogeny wide measure of dN/dS, while allowing us to determine branch-specific values of dN and dS (Additional file 1: Figure S2 and S3).

### Summary of Molecular Evolution of Germ Line Genes in Each Genus

We first summarize the patterns of $\overline{\mathrm{dN}}$/$\overline{\mathrm{dS}}$ and codon usage bias across germ line genes examined within each genus. The $\overline{\mathrm{dN}}$/$\overline{\mathrm{dS}}$ for each germ line gene across all six species branches in the *Drosophila* phylogeny and the four species in each of *Caenorhabditis* and *Apis* are reported in Tables 2, 3 and 4 (mean dN and mean dS values are provided in Additional file 1: Tables S4-S6). We found that $\overline{\mathrm{dN}}$/$\overline{\mathrm{dS}}$ varied extensively among genes within each genus. As an example, across all 34 germ line genes studied in *Drosophila* the $\overline{\mathrm{dN}}$/$\overline{\mathrm{dS}}$ values ranged from a high of 0.1573 in the LSI gene *osk* to a low of 0.0001 (Table 2) in *mago nashi (mago)*. Similar patterns were observed in the genus *Caenorhabditis*, with $\overline{\mathrm{dN}}$/$\overline{\mathrm{dS}}$ values ranging from a high of 0.1619 for the LSI gene *pie-1* to a low of 0.0081 for *mag-1*, the ortholog to *Drosophila mago*. For *Apis*, values varied from 0.0001 to 0.1393, for the ortholog to *mago* (tied with *mad* and *nos*) and *piwi* respectively. Together, these patterns show there has been marked variation in selective pressures on germ line genes in each of the three genera, and that these germ line genes are not a homogenous group sharing similar selective profiles.

**Table 2:** $\overline{\mathrm{dN}}$/$\overline{\mathrm{dS}}$ and mENC’ across the phylogeny of six species of the reference model *Drosophila*

Gene	$\overline{\mathrm{dN}}$/$\overline{\mathrm{dS}}$	Mean mENC'	SE	Alignment Length (codons)
Lineage-specific Inheritance *N*=2				
*osk*	0.1573	52.18	0.29	580
*pgc*	0.0933	37.25	0.66	71
Inheritance *N*=13				
*capu*	0.1189	54.26	0.35	1007
*orb*	0.1102	54.09	0.25	789
*stau*	0.0885	53.59	0.19	990
*cup*	0.0878	54.23	0.55	1048
*vls*	0.0810	51.02	0.44	367
*armi*	0.0773	54.28	0.44	888
*spir*	0.0751	54.34	0.17	1001
*tud*	0.0716	53.77	0.38	1446
*cycB*	0.0692	50.23	0.41	509
*gcl*	0.0392	55.13	0.20	568
*bru-1*	0.0369	51.39	0.19	723
*psq*	0.0297	49.71	0.27	469
*mago*	0.0001	42.29	0.72	147
Induction *N*=15				
*bnl*	0.1303	53.17	0.43	494
*btl*	0.0935	53.99	0.63	1040
*wg*	0.0833	47.48	0.95	261
*tkv*	0.0734	50.42	0.32	561
*byn*	0.0575	49.71	0.23	651
*Blimp-1*	0.0572	51.06	0.44	1110
*dpp*	0.0517	51.64	0.24	430
*wit*	0.0462	53.52	0.39	897
*med*	0.0422	52.47	0.57	759
*sax*	0.0402	50.88	0.80	565
*sog*	0.0305	49.45	0.48	919
*gbb*	0.0303	49.58	0.72	451
*mad*	0.0248	49.75	0.54	510
*punt*	0.0232	52.42	0.31	433
*smox*	0.0135	50.54	0.17	468
Inh/Ind *N*=4				
*nos*	0.1145	50.51	0.38	389
*vasa*	0.0545	50.59	0.70	572
*piwi*	0.0446	54.91	0.76	711
*pum*	0.0320	50.53	0.38	798

NOTE. Results are shown for each of the 34 genes under study a measured using codeml in PAML [55]. Genes are classified based on their role in PGC specification and ranked by $\overline{\mathrm{dN}}$/$\overline{\mathrm{dS}}$ within each group. The mean dN and mean dS values are provided in Table S4. SE=standard error for mENC’.

**Table 3:** $\overline{\mathrm{dN}}$/$\overline{\mathrm{dS}}$ and mENC’ across the phylogeny of four species of *Caenorhabditis*

CE Gene	DM Gene	$\overline{\mathrm{dN}}$/$\overline{\mathrm{dS}}$	Mean mENC'	SE
Lineage-specific Inheritance *N*=2				
*pie-1*	-	0.1619	46.13	0.42
*pgl-1*	-	0.1553	50.29	0.45
Inheritance *N*=8				
*cyb-2*	*cycB*	0.1127	45.01	0.68
*cpb-3*	*orb*	0.0833	51.56	1.40
*gcl-1*	*gcl*	0.0719	52.78	0.58
*ifet-1*	*cup*	0.0570	51.68	0.58
*stau-1*	*stau*	0.0499	52.63	0.80
*par-1*	*par-1*	0.0433	51.35	0.46
*etr-1*	*bru-1*	0.0386	50.54	1.52
*mag-1*	*mago*	0.0081	38.41	1.79
Induction *N*=10				
*dbl-1*	*dpp*	0.0963	51.18	0.80
*let-756*	*bnl*	0.0748	49.36	1.09
*sma-6*	*sax*	0.0675	50.00	1.30
*egl-15*	*btl*	0.0639	52.74	0.37
*blmp-1*	*Blimp-1*	0.0575	52.56	0.41
*sma-4*	*med*	0.0548	52.34	0.67
*tig-2*	*gbb*	0.0522	50.24	1.15
*crm-1*	*sog*	0.0395	47.73	0.45
*cwn-1*	*wg*	0.0264	49.69	0.42
*sma-2*	*mad*	0.0128	46.30	0.78
Inh/Ind *N*=3				
*glh-1*	*vasa*	0.0761	43.98	1.48
*puf-8*	*pum*	0.0753	50.79	0.92
*prg-1*	*piwi*	0.0642	46.13	1.81

NOTE. Values are shown for each of the 23 genes under study as measured using codeml in PAML [55]. Genes are ranked from highest to lowest $\overline{\mathrm{dN}}$/$\overline{\mathrm{dS}}$ values within each category. *CE Caenorhabditis elegans*, *DM D. melanogaster*. The inheritance gene *par-1* was included for *Caenorhabditis* (see Methods). *SE* standard error for mENC’.

**Table 4:** $\overline{\mathrm{dN}}$/$\overline{\mathrm{dS}}$ and mENC’ across the phylogeny of four species of *Apis*

Gene	$\overline{\mathrm{dN}}$/$\overline{\mathrm{dS}}$	Mean mENC'	SE
Inheritance N=13			
*armi*	0.1011	54.66	0.13
*tud*	0.0792	56.67	0.27
*cycB*	0.0774	53.24	0.07
*vls*	0.0673	51.78	0.34
*spir*	0.0564	56.22	0.05
*bru-1*	0.0560	53.13	0.15
*stau*	0.0395	51.24	0.10
*gcl*	0.0326	53.42	0.27
*capu*	0.0238	53.39	0.53
*orb*	0.0192	55.04	0.13
*psq*	0.0173	54.07	0.32
*par-1*	0.0067	54.84	0.25
*mago*	0.0001	46.43	0.39
Induction N=13			
*dpp*	0.1342	52.24	0.14
*sax*	0.1163	51.80	0.24
*wg*	0.0854	48.68	0.48
*sog*	0.0627	55.22	0.21
*wit*	0.0462	55.48	0.15
*Blimp-1*	0.0375	49.42	0.72
*gbb*	0.0363	46.37	0.40
*punt*	0.0335	52.73	0.39
*byn*	0.0324	53.20	0.25
*med*	0.0133	52.72	0.26
*tkv*	0.0116	53.91	0.09
*smox*	0.0080	52.44	0.36
*mad*	0.0001	51.63	0.29
Inh/Ind *N*=4			
*piwi*	0.1393	54.72	0.39
*vasa*	0.1155	53.33	0.31
*pum*	0.0038	53.84	0.29
*nos*	0.0001	38.89	0.96

NOTE. Values are from codeml in PAML [55]. Genes are listed using the ortholog name from *D*. *melanogaster*. The inheritance gene *par-1* was included for *Apis* (see Methods). *SE* standard error for mENC

In addition to $\overline{\mathrm{dN}}$/$\overline{\mathrm{dS}}$, we measured codon usage bias (CUB) for each of the germ line genes within each genus. The effective number of codons (ENC) determines the deviation from equal usage of all codons in a gene, wherein values range from 20 to 61 (number of codons in the genetic code), and lower values indicate greater codon usage bias [60]. We used the modified ENC’ measure (mENC’), which accounts for abundance of rare amino acids and for nucleotide content (mutational biases) of the genes under study [61, 62]. Preferential usage of codons is thought to usually (but not always) result from weak but persistent selection pressures that promote efficient and accurate transcription and/or translation, as has been reported in studies of *Drosophila*, *Caenorhabditis* and *Apis* [63–68].

The results showed that of all 34 germ line genes studied in *Drosophila*, the highest and lowest CUB were observed in the two LSI genes, *pgc* and *osk* respectively (mENC’=37.25±0.66 and 52.18±0.29 respectively, values are means and standard errors across species, Table 2). This result reveals marked differences in the degree of codon usage bias of the two LSI genes. In turn, 25 of the remaining 32 studied genes exhibited mENC’ values that were >50, implying comparatively low CUB, with respect to *pgc*. For the genus *Caenorhabditis*, among the 23 germ line genes studied in that genus, the CUB was highest for *mag-1* (mENC’=38.41±1.79) and lowest for *gcl-1* (52.78±0.58), with intermediate values observed for its two LSI genes (Table 3). The broadest range of CUB was observed within the genus *Apis* where mENC’ values ranged from 38.89±0.96 to 56.67±0.27 (Table 4), suggesting a propensity for greater variation in CUB of germ line genes in that taxon.

In sum, it is evident that germ line genes exhibit wide variation in selective pressures on protein sequence divergence and in codon usage bias within each of the three genera under study here (see the below section “*Relative Ranking of dN/dS and CUB Between Genera*” for details on how dN/dS and CUB compared between genera). In this regard, the germ line genes are not a homogenous group exhibiting a similar range of $\overline{\mathrm{dN}}$/$\overline{\mathrm{dS}}$ values or common CUB profiles. Sex and reproductive gene proteins are thought to often evolve rapidly, particularly those involving gametogenesis and sperm and eggs [69, 70]. However, our results showed that a subset of germ line genes had $\overline{\mathrm{dN}}$/$\overline{\mathrm{dS}}$ <0.05, which suggests very high purifying selection (Tables 2, 3 and 4), and thus that pattern may not broadly apply to genes involved in germ line specification or development (see also, [50, 51]). Nonetheless, certain germ line genes studied here, such as the LSI genes, exhibited comparatively rapid evolution, suggesting they may be particularly significant to the evolutionary changes of the molecular mechanisms regulating germ lines in each genus.

### Rapid Evolution of LSI Genes in *Drosophila* and in *Caenorhabditis*

#### Accelerated Divergence of LSI Genes in Drosophila

The LSI germ line genes are of particular interest as they may be crucial to enhancing our understanding the evolution of germ plasm in animals, since they appear to be *de novo* genes that have arisen and developed specialized germ line functions within only certain animal lineages. Our results show that the proteins of LSI genes are among the most rapidly evolving of the 34 germ line genes under study in *Drosophila*. Specifically, the finding that the highest $\overline{\mathrm{dN}}$/$\overline{\mathrm{dS}}$ in *Drosophila* was observed for the LSI gene *osk* (0.1573, Table 2) suggests it may have experienced the least constraint among all the studied germ line genes in this genus. The *osk* gene is involved in germ plasm assembly and has only been reported to date in *Drosophila* and certain insects [1, 2, 4]. Osk proteins are essential for recruitment of molecules to germ plasm (e.g. via direct interactions with *Vasa* and *Staufen* proteins) and have RNA-binding functions to *nos* and its own mRNA [18, 42, 71, 72]. The $\overline{\mathrm{dS}}$ value for *osk* was intermediate (0.2779), near the median of values across all germ line genes (0.2580; Additional file 1: Table S4). However, its $\overline{\mathrm{dN}}$ value (0.0437) was the highest observed across all studied genes (Additional file 1: Table S4), affirming that its elevated $\overline{\mathrm{dN}}$/$\overline{\mathrm{dS}}$ value is due to accelerated protein sequence divergence. Such high nonsynonymous changes, if not adaptive, would be apt to often be deleterious in a gene essential for fecundity and fitness [73], and thus would be unlikely to be fixed. In this regard, the high $\overline{\mathrm{dN}}$/$\overline{\mathrm{dS}}$ in *osk* appears potentially to be the result of episodic adaptive evolution. That is, changes in amino acids of protein sequences may have been retained due to positive selection, via a selective advantage of the phenotypes associated with these protein sequence changes [55, 74–76]. Analysis of positive selection using sites analysis in PAML (see Methods, [55]), which we used to test for positive selection at specific codon sites in each gene across all six *Drosophila* species, did not show positive selection within this LSI gene (Table 5). This is similar to results reported in a prior assessment of the gene *osk* within the *melanogaster* group [52] (the previously reported sites under putative positive selection were reported from a branch site test for *D. virilis*, a species outside of the *melanogaster* group and thus not studied herein [52]). However, positive selection analyses (in specific branch-sites or sites) can be highly conservative [56, 76, 77], and often lack sensitivity to detect functional changes [78]. In this regard, positive selection cannot be excluded by the absence of statistically significant sites tests.

**Table 5:** Results from sites analysis of positive selection (M7 versus M8) in *Drosophila*, *Caenorhabditis* and *Apis*

Gene	Role		*P*	Sites (BEB Probabilities)
*Drosophila*				
*byn*	Induction		**	29 S**
*capu*	Inheritance		**	252 P**, 253H**, 437 T*
*Caenorhabditis*				
*cpb-3*	Inheritance		**	423 N*
*let-756*	Induction		**	231 S*
*par-1*	Inheritance		**	468 A**, 625 N*, 647 Q*, 701 G**, 704 T**,705V*
*Apis*				
*bru-1*	Inheritance		**	337 T*, 339 A*, 341 A*
*cycB*	Inheritance		**	4 G**, 5 L**
*dpp*	Induction		*	80 S*, 81 T*, 86 Q**, 87 L *
*med*	Induction		**	-
*orb*	Inheritance		**	-
*piwi*	Inh/Ind		**	153 H**,318 T** ,320 A **
*psq*	Inheritance		**	-
*pum*	Inh/Ind		**	306 Y*
*stau*	Inheritance		**	-
*wit*	Induction		**	329 I*

NOTE: Analysis was conducted in PAML [55]. Only those genes at or near statistical significance are shown. ** *P*<0.05 for 2X∆lnL *P=0.062. BEB probabilities for specific sites are indicated as ** *P*≥0.95 (**) and *0.95≥*P*>0.9

Here, based on several lines of evidence we propose that adaptive evolution may have contributed towards the observed rapid protein sequence evolution of *osk*, as compared to all other 33 *Drosophila* germ line genes studied herein (Table 2). First, experimental findings have shown that functionality of *osk* has evolved rapidly, namely based on an inability of the gene in one *Drosophila* species (*D. virilis*) to rescue loss of function mutations in *D. melanogaster* [79], although these two species shared a last common ancestor > 55 My [80]. Second, some functional binding regions within the gene have been shown to have at least two-fold higher dN/dS than other segments [52], which may be deemed consistent with non-random, and thus putatively adaptive, changes. Third, relaxed selection appears unlikely to explain the relatively fast evolution of *osk* given that this lineage-restricted *de novo* gene has evolved crucial functions, involving high protein and RNA interactivity, in the germ plasm and during PGC-specification [2, 4, 18, 42, 71, 72]. These functions may act as constraints that limit relaxed purifying selection. Given that *osk* evolved before the advent of germ plasm in insects [81], such that the ancestral role of *osk* was unlikely to have been be a germ plasm role, we can infer that the evolution of essential roles in germ plasm must have been due to changes that arose following its origin approximately 300 Mya [79, 81], and such episodic changes may have been ongoing in the ~44 Mya history of the melanogaster group of *Drosophila* studied here [80]. Fourth, adaptive changes linked to germ line functions may have been facilitated by the low expression breadth observed for *osk* across development (as shown in the below section, “Expression breadth and pleiotropy in *Drosophila*”). Low pleiotropy appears characteristic of functional *de novo* genes [21, 82, 83], and while this might sometimes cause relaxed selection, it can also allow adaptive protein changes with minimal interference from functions in other tissues [84]. Collectively, these four lines of evidence suggest that the rapid divergence of *osk* observed here is at least partly the result of a history of episodic adaptive evolution within the *Drosophila* genus.

The other *Drosophila* LSI gene, *pgc*, also evolved relatively rapidly compared with all 34 *Drosophila* genes studied ($\overline{\mathrm{dN}}$/$\overline{\mathrm{dS}}$ = 0.0933, ranked 7^th^ of 34 genes). *pgc* encodes a small protein (72 codons in *D. melanogaster*, Table 1) that is involved in transcriptional silencing in PGCs by preventing phosphorylation of RNA PolII and by inhibiting recruitment of the positive transcription elongation factor b (P-TEFb) to transcription sites. The suppression of expression is crucial for preventing germ cells from differentiating into somatic cells, and thus for sustaining the germ line [19]. Our alignment for six species is nearly identical to that produced by Hanyu-Nakamura et al. [19] who studied functionality of this protein in *Drosophila* (including more divergent taxa) and suggested that it is highly conserved. Our findings showing $\overline{\mathrm{dN}}$/$\overline{\mathrm{dS}}$ of 0.0933, which is <<1, concurs with conservation, but nonetheless, indicates that with respect to other genes involved in germ line development, this protein evolves notably rapidly. We note that we examined the dN/dS of all orthologous protein-coding genes in the Drosophila clade studied here using the data provided by the flyDIVaS resource [57], and found that *pgc*, like *osk*, was above the average observed across the genome (see Additional file 1: Text file S1). As *pgc* is believed to be restricted to *Drosophila* and is not found in other metazoans, including within fellow Dipteran insects [19], its *de novo* status as a germ plasm regulator, similar to *osk*, appears to be a factor potentially shaping its fast divergence as compared to most other germ line genes.

With respect to codon usage, as described earlier the mENC’ value was extremely low for *pgc* in *Drosophila* (37.25±0.66, Table 2), in fact lowest among all 34 genes studied in this insect and was comparatively higher in *osk* (52.18±0.29; Mann Whitney U-test *P*<0.002), suggesting CUB is under greater selective constraint in *pgc*. This is also supported by higher GC3s in *pgc* than *osk* (mean values of 0.712±0.015 and 0.652±0.010 respectively, MWU-test P=0.004, Additional file 1: Table S7), indicating greater use of optimal codons, which have been shown to typically end in G and C in these *Drosophila* taxa [66, 85]. Thus, the greater CUB in the former gene might reflect a crucial role of efficient translation of this gene, potentially due to high translation rates during PGC formation. Alternatively, it might also be connected to the exceptionally short CDS length of *pgc,* which comprises the shortest CDS under study (Table 1), a feature which has been proposed to be linked to elevated selection coefficients on codon usage [86] and to be associated with greater CUB [63, 66].

#### Accelerated Divergence of LSI genes in Caenorhabditis

With respect to the nematode genus *Caenorhabditis*, we studied two LSI genes, *pie-1* and *pgl-1*, each of which showed markedly elevated $\overline{\mathrm{dN}}$/$\overline{\mathrm{dS}}$ among the 23 germ line genes under investigation in this genus (Table 3). For instance, as shown in Table 3, *pie-1* and *pgl-1* in *Caenorhabditis* had the two highest $\overline{\mathrm{dN}}$/$\overline{\mathrm{dS}}$ values of all 23 germ line genes under study (0.1619 and 0.1553). This suggests that, similar to the situation in *Drosophila*, genes specifically involved in germ plasm, and that arose independently within a lineage, have experienced accelerated divergence as compared to other germ line genes. *pie-1* and *pgl-1* products are localized to the P granules and essential to fertility, have not been reported in other metazoans and are thought to be specific to *Caenorhabditis* [3, 4, 22, 29, 87, 88]. PIE-1 is crucial to germ line establishment as it represses mRNA transcription in germ line blastomeres and prevents differentiation into somatic cells by inhibiting activity of P-TEFb, which ultimately impedes phosphorylation of RNA PolII and prevents transcription elongation [19, 87, 89]. In this regard, *pie-1* shares functionality (but not sequence homology) with the rapidly evolving *Drosophila* gene *pgc* (Tables 2 and 3) [19]. Remarkably, our data here shows that *pie-1* and *pgc,* which are *de novo* genes with convergent functions that have arisen independently in *Caenorhabditis* and in *Drosophila* respectively [4, 19], each diverge rapidly as compared to other germ line genes in their associated taxonomic group (Tables 2 and 3).

The relatively rapidly diverging *Caenorhabditis* LSI gene *pgl-1* is essential in the P granule assembly pathway [3, 88, 90]. Its protein contains RGG-binding motifs (which are linked to genes involved in transcription, translation, splicing) similar to those found in the protein product of *vasa* (*glh-1* in *Caenorhabditis*) [3, 22]. The P granule pathway presumably involves the localization of *pgl-1* products to the P granules by *glh-1*, as mutants of the latter gene contain PGL proteins dispersed throughout the cytoplasm [3]. We found that *glh-1* had a $\overline{\mathrm{dN}}$/$\overline{\mathrm{dS}}$ of 0.0761, which is about half the value of *pgl-1* (0.1553). This suggests that *pgl-1* has evolved at a much faster rate than its localization protein, whilst presumably not interfering negatively with their interaction. While *pgl-1* is part of a gene family with other members *pgl-2* and *pgl-3* that may have arisen from gene duplication, the other paralogs are, unlike *pgl-1*, not essential to P granules or PGCs, as *pgl-2* and *pgl-3* loss of function mutants do not exhibit obvious defects in germ line development on their own [3, 90]. *pgl-3*, however can be partially redundant to *pgl-1* under cold temperatures [3, 90]. Thus, the rapid evolution of *pgl-1* might partly result from some redundancy (or relaxed selection) of function under specific conditions. However, the fact that the other LSI genes studied in *Drosophila* and in *Caenorhabditis* do not have apparent paralogs, and each evolve relatively fast among the germ line genes (Tables 1 and 2), suggests that accelerated protein divergence is a common feature of the lineage-restricted germ plasm genes, rather than being an artefact due to the existence of a partially redundant paralog of *pgl-1*.

With respect to CUB, *pie-1* exhibited greater bias (mean mENC’ 46.13±0.42) than *pgl-1* (50.29±0.45; MWU test P=0.029), suggesting enhanced selection on CUB in the former gene, perhaps reflecting a higher translation rate. However, similar to *pgc* from *Drosophila*, the short CDS of *pie-1* might contribute to its high CUB. Short CDS not only generally exhibit high CUB, but sometimes also low dN/dS (and/or dN), trends perhaps mediated by protein-protein interactions and/or elevated expression levels [63, 86, 91, 92]. Whilst *pie-1* is markedly shorter than *pgl-1* (note the average lengths per gene across species were 351±8,9 and 770±4.8 codons, a nearly two-fold difference; the conservative alignment lengths had nearly three-fold difference) consistent with elevated CUB, the $\overline{\mathrm{dN}}$/$\overline{\mathrm{dS}}$ values were very similar between genes (Table 3), thus suggesting while length might influence the relative CUB, it is unlinked to their amino acid divergence in *Caenorhabditis*. Alternatively, it is possible that adaptive evolution at specific amino acid sites has occurred more frequently for *pgl-1*, leading to selective sweeps at linked sites containing slightly deleterious non-optimal codon mutations [93], which over the long-term can reduce CUB [93, 94]. Although positive selection was not found in the four *Caenorhabditis* species studied here for these two genes using sites analysis (Table 5), further studies including even more species, as data becomes available, will allow greater power of these tests and fuller discernment of the role of positive selection in these genes.

In our assessment, we also wished to examine the *Caenorhabditis* LSI gene *meg-1*, which is a P-granule component required for germ line development [27], but found that identification of *meg-1* orthologs for all four species was ambiguous. The best matches to the *C. elegans meg-1* had an e-value of 5.0X10^-5^ for *C. brenneri,* 6.0X10^-4^ for *C. briggsae* and 0.053 for *C. remanei*, and were largely unalignable across most of the sequence. We therefore excluded this gene from analysis herein. The lack of clearly identifiable orthologs is suggestive of rapid divergence or potential gene loss in nematodes, or might indicate that this *de novo* gene occurs solely in a single species, *C. elegans* [27]. While *meg-1* is an LSI gene, multiple copies (*meg-1-4*) have been reported in *C. elegans* with partial overlap in function [28], and this might explain potential rapid sequence evolution and/or gene losses in some species of this genus.

#### Summary of Findings on LSI Genes

Taken together, the collective results from LSI genes from *Drosophila* and *Caenorhabditis* (*osk*, *pgc*, *pie-1* and the *pgl-1*) suggest all these *de novo* genes evolve rapidly as compared to other studied germ line genes. As these four genes appear not to have originated from a gene duplication due to the absence of ancestral orthologs [whilst *pgl* has multiple paralogs in Caenorhabditis, *pgl* genes appear limited to nematode genomes; 3], we speculate that these LSI protein-coding genes might have arisen at least partially from noncoding regions [95–98] or horizontal gene transfer [21, 99]. In fact, recent evidence has supported a putative role of horizontal gene transfer in the origin of *osk* [100]. *De novo* genes have been previously linked to expression in sexual organs, including germ lines [21, 95, 96]. Nonetheless, whilst it appears these LSI genes have not arisen from duplication, we can neither formally exclude nor directly test the hypothesis that they originally arose from a duplication and evolved so rapidly that the orthologs cannot be identified [21].

Regardless of the precise origin, these four *de novo* genes have not degenerated into pseudogenes or been loss from the genome due to lack or loss of function, as frequently occurs for orphan genes [101], but rather play a crucial role in PGC specification and thus fertility. A plausible explanation for their existence and evolution of novel functionalities is their involvement in lineage-specific adaptive processes [102], potentially accompanied by phenotypic novelties, as is thought to occur for *de novo* genes that become functional [98, 102, 103]. For LSI genes, the adaptations would involve their crucial roles in germ plasm, which is believed to be a novelty in the context of nematodes and insects [7]. This notion is further consistent with recent findings in *Drosophila* that surviving (not lost from the genome) *de novo* genes exhibit functionalities specific to a narrow developmental phase or tissue type [82]: germ plasm and the PGCs are limited to the stages involving the egg or early embryo. It can be speculated that adaptive amino acid changes in LSI genes might have been historically mediated by sexual selection, as the pre-formed germ plasm could conceivably indirectly influence sperm-egg fertilization success (and thus, sexual antagonism), from natural selection on germ plasm due to its effect on zygotic or embryonic fitness, and/or from cell-lineage selection among the precursors to PGCs or among PGCs, each of which could accelerate protein sequence divergence [30].

We speculate that a history that includes episodic adaptive evolution in the emergence of functions in the *de novo* LSI genes may have potentially continued after the establishment of their primitive germ plasm roles and extended to within the intra-genus level. This notion appears consistent with multiple lines of evidence for *osk* (Table 2) [52, 79], and could account for elevated $\overline{\mathrm{dN}}$/$\overline{\mathrm{dS}}$ observed for all four of the LSI genes studied here (Tables 2 and 3). Nonetheless, we do not exclude a role of neutral functional or non-functional amino acid changes in the rapid divergence of LSI genes relative to other germ line genes, which appears consistent with the absence of detection of positive selection in Table 5; however, such tests can be highly conservative [77] and be prone to substantial inaccuracies [104, 105] (see Additional file 1: Text file S2), and thus cannot reliably exclude positive selection. At present, much remains to be unknown about the evolution of *de novo* functional genes, including about the roles of adaptive and neutral changes in those genes that that form essential roles in genetic networks [21, 97, 98], such as has occurred for the LSI genes involved in germ plasm.

### Expression Breadth and Pleiotropy in *Drosophila*

Protein sequence evolution may be influenced by pleiotropy, where the greater a gene’s involvement in multiple functions or tissues, the more indispensable it may be to fitness, and thus more likely to exhibit stringent purifying selection [83, 84, 106]. In turn, those genes with reduced pleiotropy may be more dispensable and evolve faster due to relaxed selective pressures (neutral changes), and/or due to adaptive functional changes unimpeded by pleiotropic constraints [84, 107]. Expression breadth across developmental stages and tissues provides a proxy for the pleiotropy of a gene [83, 84, 106]. Further, data from Drosophila suggests that young *de novo* gene products which exhibit functional roles may often have those roles restricted to one or a few developmental stages, and thus may be specialized for specific developmental processes [82]. We thus determined expression breadth across developmental stages/tissues with expression using the large-scale transcriptome data available for our main target and reference taxon *Drosophila*, from its most well studied model species, *D. melanogaster* [48, 108, 109]. For each of the 34 *Drosophila* germ line genes in Table 2, we measured expression breadth across 30 developmental stages (spanning from 0-2 hour embryos, larvae, pupae, to adult males and females, described in Additional file 1: Table S8).

**Fig. 1.**
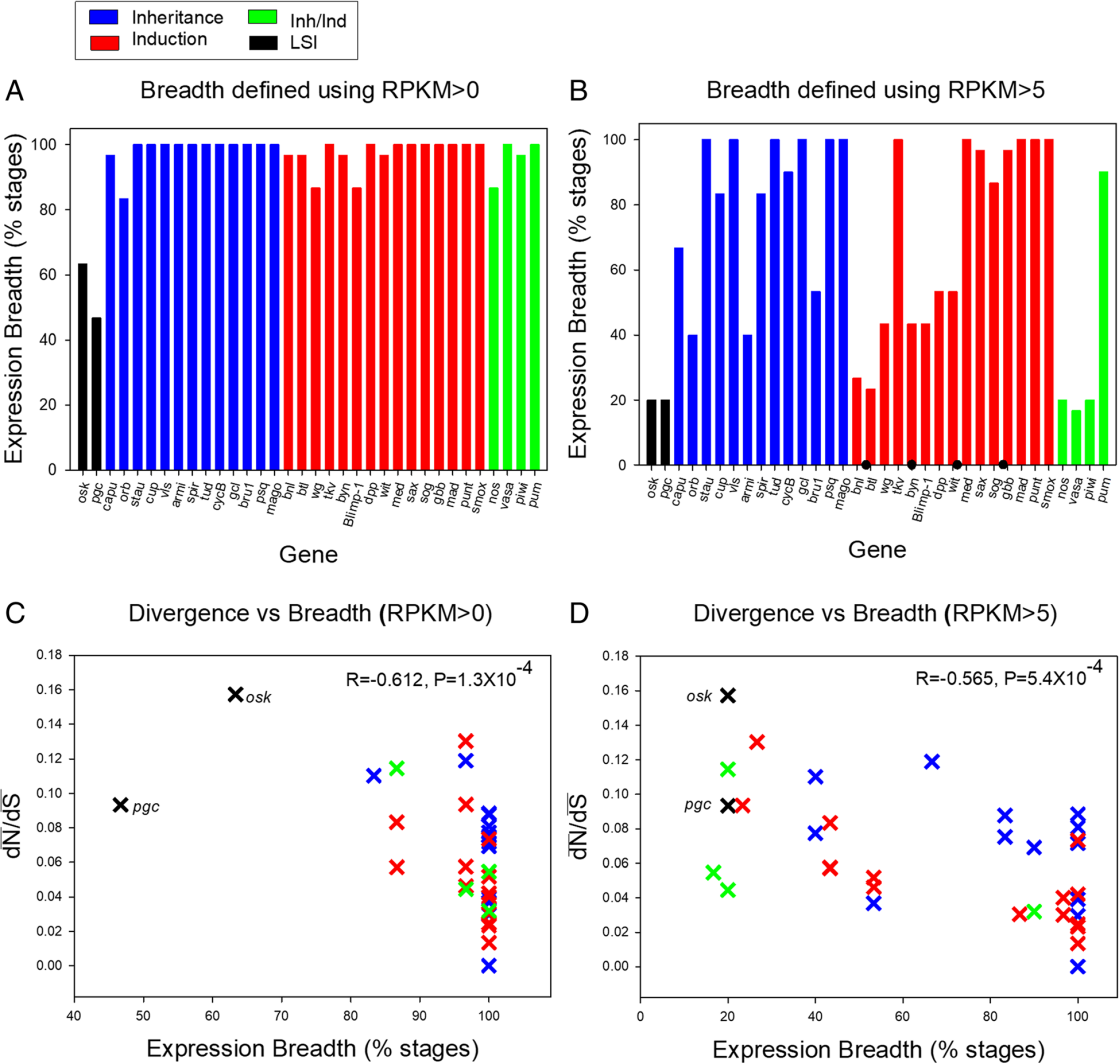
The expression breadth (percentage of 30 developmental stages expressed) for the 34 *Drosophila* genes under study and its relationship to protein divergence. **a** breadth of expression across developmental stages at a level >0 RPKM; **b** breadth of expression at a level >5 RPKM; **c** $\overline{\mathrm{dN}}$/$\overline{\mathrm{dS}}$ versus expression breadth (RPKM>0); **d** $\overline{\mathrm{dN}}$/$\overline{\mathrm{dS}}$ versus expression breadth (RPKM>5). The 30 tissues and stages are provided in Table S8. For A-B, genes are listed on the X-axis in the same order as presented in Table 2, from highest to lowest $\overline{\mathrm{dN}}$/$\overline{\mathrm{dS}}$ within each of the four categories of genes. Gene names associated with each dN/dS point are provided in Table 2.

As shown in Fig. 1a, we found that the majority of germ line genes in Table 2 (*N*=22 of 34) were expressed ubiquitously at a level of >0 RPKM in all the disparate stages (expression breadth=100%; *N*=28 had values of >95%), indicating high pleiotropy in these germ line genes. The LSI genes *osk* and *pgc* had the lowest values, with a breadth of 63.3 and 46.7% respectively. Using a higher cutoff of >5 RPKM to define specificity (Fig. 1b), we observed even more variation in expression breadth; most genes had values between 40-100%, but remarkably low breadth was observed for Inh/Ind genes such as *vasa* and *piwi* and again for the two LSI genes, which had values of 20% (Fig. 1b). With respect to $\overline{\mathrm{dN}}$/$\overline{\mathrm{dS}},$, we observed a negative correlation between expression breadth and $\overline{\mathrm{dN}}$/$\overline{\mathrm{dS}}$ using both criteria of >0 RPKM and >5 RPKM (Spearman R=-0.612, P=1.3X10^-4^ and R=-0.565, P=5.4X10^-4^ respectively, (Fig. 1c and d) consistent with higher protein divergence in narrowly expressed genes. In contrast, no correlation was observed between $\overline{\mathrm{dN}}$/$\overline{\mathrm{dS}}$ and the average expression level across developmental stages (P=0.926). mENC’ was uncorrelated to expression breadth (P=0.498). Together, these data suggest that most of the germ line genes under study exhibit broad expression, or high pleiotropy. In turn, low expression breadth may substantially contribute towards the accelerated evolution of the LSI genes.

As an example, the extraordinarily low expression breadth for the LSI genes *osk* and *pgc*, using both the criteria of >0 RPKM (Fig. 1a and c) and >5 RPKM (Fig. 1b and d), indicates high specialization and limited pleiotropy of those genes, consistent with their specialized functions in germ plasm. For both genes, the only stages with relatively high expression (for instance, using a cut-off of >15 RPPM) were adult females (42 to 372 RPKM) and 0-4 hour embryos (17 to 270 RPKM), consistent with their PGC specification roles in female sexual cells and young embryos. This observed pattern concurs with the notion that young *de novo* genes exhibit expression limited to one or a few developmental stages as they acquire new functions in an organism [21, 82]. Further, such *de novo* genes may become essential if they integrate into existing networks [21], as appears to be the case for *osk* and *pgc* where these genes have assumed an upstream regulatory role in the pathway of PGC-specification and germ line development [4, 17]. Moreover, their low pleiotropy may facilitate evolution of new functions by permitting adaptive evolution largely unimpeded by co-functionality in other tissues [84], and/or may contribute towards divergence via drift (see above section “Rapid evolution of LSI genes in *Drosophila* and in *Caenorhabditis*”). Further consideration of the putative role of pleiotropy with respect to the $\overline{\mathrm{dN}}$/$\overline{\mathrm{dS}}$ of specific germ line genes is provided in Additional file 1: Text File S3.

### Relative Ranking of dN/dS and CUB Between Genera

After considering the evolution of LSI genes, we next asked whether the germ line genes studied here shared parallels in their patterns of molecular evolution across genera. For this, we assessed whether the relative ranking of $\overline{\mathrm{dN}}$/$\overline{\mathrm{dS}}$ and of mENC’ were similar between our reference model *Drosophila* and *Caenorhabditis* and *Apis*. By examining the relative ranking of dN/dS and of CUB, between genera using Spearman’s Ranked R (not absolute values), this controls for taxon-specific factors, and allows us to assess if the relative dN/dS and CUB within this group of germ line genes has been retained across genera.

**Fig. 2.**
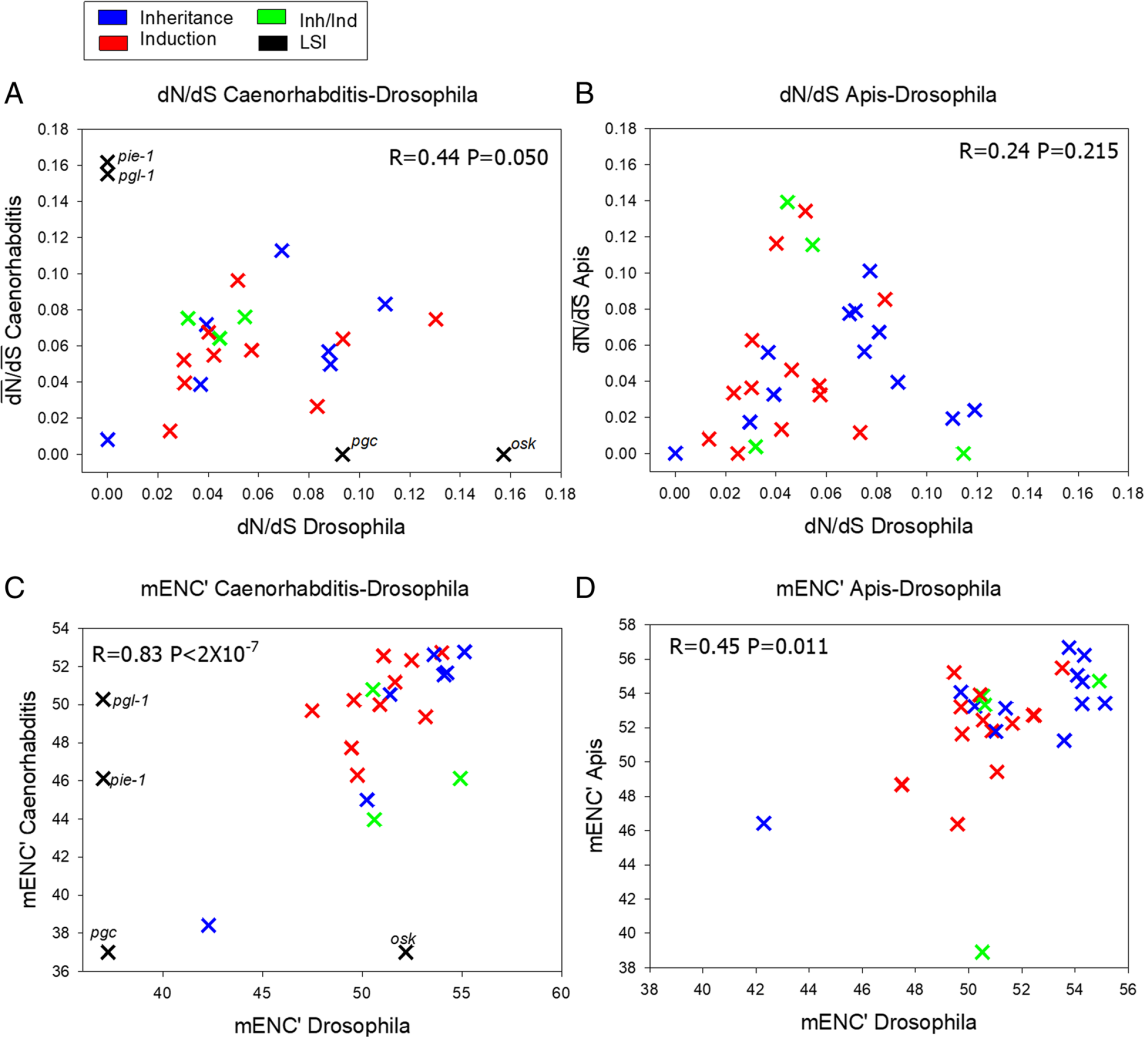
Correlations in $\overline{\mathrm{dN}}$/$\overline{\mathrm{dS}}$ and in mENC’ between *Drosophila* and *Caenorhabditis* and *Drosophila* and *Apis*. **a** The $\overline{\mathrm{dN}}$/$\overline{\mathrm{dS}}$ values for *Caenorhabditis* versus those from *Drosophila*; **b** the $\overline{\mathrm{dN}}$/$\overline{\mathrm{dS}}$ values for *Apis* versus those from *Drosophila*; **c** mENC’ for *Caenorhabditis* versus *Drosophila*; **d** mENC’ for *Apis* versus *Drosophila*. Spearman’s ranked R and P values are shown across all genes for the between genus orthologs (pooled across categories). The lineage-specific inheritance (LSI) genes in *Drosophila* and in *Caenorhabditis* genes are shown on the X and Y axis respectively in A and C and were not included in the calculation of between genus correlations. The gene names associated with each value of $\overline{\mathrm{dN}}$/$\overline{\mathrm{dS}}$ and mENC’ shown are provided in Tables 2, 3, and 4.

As shown in Fig. 2, for the *Drosophila* genes having orthologs identified in *Caenorhabditis*, we found $\overline{\mathrm{dN}}$/$\overline{\mathrm{dS}}$ was positively correlated between genera (pooled across all categories, excluding the LSI genes, Spearman’s rank correlation R=0.44, P=0.05, N=20, Fig. 2a). However, the correlation was not statistically significant for the orthologs between *Drosophila* versus *Apis* (P=0.215, Fig 2b; note, nor for those common to *Caenorhabditis* vs. *Apis* (*P*=0.112)). Reducing the *Drosophila*-*Apis* ortholog gene list (which was larger than for the distant nematodes, Fig. 2, Tables 3 and 4) to those also found in *Caenorhabditis*, also did not yield a correlation between *Drosophila* and *Apis* (*P*=0.181). Thus, this indicates that the germ line gene sets share greater similarity in the relative protein divergence between *Drosophila* and its distant relative *Caenorhabditis* (from different phyla) than between the two insects. The values of $\overline{\mathrm{dN}}$/$\overline{\mathrm{dS}}$ in *Drosophila*, *Caenorhabditis* and *Apis* were in a largely similar range across all the three genera examined here (Tables 2, 3 and 4, Fig. 2). Thus in that context the magnitude of $\overline{\mathrm{dN}}$/$\overline{\mathrm{dS}\ }$ did not appear to vary among taxa, but rather the relative dN/dS among genes simply appeared more conserved between *Drosophila* and *Caenorhabditi*s than either was to *Apis*. Previous reports have shown that *Drosophila* and *Caenorhabditi*s, despite being from different phyla, share striking parallels in gene expression profiles and networks across developmental stages [108]. In this regard, our present data suggest these divergent systems also share similar relative protein divergence patterns of their germ line genes. One obvious similarity between these organisms with respect to germ lines that speculatively could contribute towards this latter pattern is the use of the inheritance mode in both models. However, further study in more genera would be needed to ascertain whether specification mode plays any role in these shared patterns.

In terms of CUB, we report a strong positive correlation between mean mENC’ of genes in *Drosophila* and their orthologs in *Caenorhabditis* (*R*=0.83, *P*=2X10^-7^) and also a significant positive correlation between *Drosophila* and *Apis* (R=0.45, P=0.011) (Fig. 2c and d). This suggests the relative CUB in each germ line gene set has been largely retained across these disparate organisms, particularly between the flies and nematodes. We note that the mENC’ levels were fairly high for many germ line genes (values >50 for genes), indicating that these germ line genes as a group do not exhibit exceptionally strong codon bias. Nonetheless, the correlation in CUB values between genera shows that the relative degree of bias tends to be largely conserved across these three divergent animal models. As CUB is believed to often promote translational efficiency of highly translated genes [63], we speculate that germ line genes might have retained their relative translation rates across divergent models.

While the nucleotide composition in germ line genes differed among taxa, including a GC bias in *Drosophila* (GC content across all germ line genes=0.561±0.006) and AT biases in *Caenorhabditis* and *Apis* (GC content=0.450±0.020 and 0.405±0.002) (Additional file 1: Table S7), nucleotide content has been accounted for using mENC’ [61, 62], and thus Fig. 2 c and d suggests that the relative selective pressure on CUB, despite different types of background nucleotides or optimal codons in these three genera [54, 56, 64, 66, 67, 85, 110] is at least partly retained across orthologous gene sets. The relevancy of correcting for background composition [61] was demonstrated by the fact that traditional ENC showed no correlation between taxa (*Drosophila* and *Caenorhabditis* P=0.392, *Drosophila* and *Apis* *P*= 0.116, Additional file 1: Figure S4).

### Protein Sequence Divergence and the Transition to Inheritance Mode

As a final note, we briefly mention here that a prior hypothesis in the literature had suggested that the transition from induction to inheritance mode results in a release of selective constraint, and accelerated evolution of proteins that is detectable at the genome-wide level [111]. We previously assessed that hypothesis using methods adhering to established principles of molecular evolution, and found some examples disagreeing with its predictions on protein sequence evolution [30, 54]. We had noted that the hypothesis may apply to smaller subsets of genes such as germ line genes, or PGC-specification genes [30, 54]. We thus compared dN/dS of *Drosophila* and *Apis,* which are each from the class Insecta, and have inheritance and induction mode respectively. We did not observe evidence consistent with accelerated evolution (or release of constraint) on proteins of germ line genes of *Drosophila* as compared to *Apis* (Tables 2 and 4, Fig. 2). For instance, dN/dS was not statistically significantly higher for *Drosophila* than for *Apis* using Mann-Whitney (Ranked) U test of all germ line genes with orthologs between these genera (*P*=0.320), or for the subset of genes with known roles under “Induction” mode (*P*=0.720). If the PGC-specification hypothesis, as it pertains to protein sequence evolution [111], indeed applied to germ line genes then a tendency towards higher dN/dS would be expected in flies after a transition to inheritance, which is not what we observed. Nonetheless, as this pattern is solely from two genera, we consider it alone anecdotal rather than conclusive or generalizable. Further study across more germ line genes and taxon groups, including even more closely related genera, would be valuable for rigorous testing of any such general relationship.

## Conclusions

Our results herein showed that germ line genes exhibit a wide range of $\overline{\mathrm{dN}}$/$\overline{\mathrm{dS}\ }$ values and CUB in each of three genera, *Drosophila*, *Caenorhabditis*, and *Apis*. Relative to other germ line genes, we found evidence that LSI genes in *Drosophila* (*osk*, *pgc*) and in *Caenorhabditis* (*pie-1*, *pgl-1*) have diverged especially rapidly, and we conclude this could be a common property of *de novo* germ plasm genes. Whilst adaptive evolution is a strong candidate to explain this fast divergence of LSI genes, particularly for *osk* which has several lines of evidence consistent with a history of positive selection in *Drosophila*, we do not exclude some role of relaxed purifying selection; both adaptive changes and relaxed selection may be facilitated by the narrow expression breadth and low pleiotropy of LSI genes, as found using data from *Drosophila*. Our findings further show that the relative ranking of $\overline{\mathrm{dN}}$/$\overline{\mathrm{dS}\ }$and of CUB germ line genes in the reference *Drosophila* were each correlated to their orthologs in *Caenorhabditis* while only CUB was correlated to the orthologs from *Apis*. The molecular evolutionary patterns of germ line genes in the flies and nematodes may be similar to developmental expression profiles [108], wherein striking parallels were observed across these organisms, despite being from different phyla.

Future research should explore how LSI genes evolve within populations, including the study of their amino acid mutational spectra relative to other identified (non-germ line) *de novo* genes [82], and to experimentally assess shifts in in their functionality within or between genera (cf. [79]). Moreover, transcriptome data from the germ lines during early embryogenesis and germ line development in multiple species per genus may provide a means to assess how their gene expression has evolved between species, which may be as relevant to understanding divergence in their function as protein sequence changes. Furthering our understanding of the molecular evolution of germ line genes will be facilitated by expanding research to species from a wider range of genera, including the induction model systems mice, crickets and salamanders [5, 8, 10, 32, 112] and inheritance species such as wasps and frogs [2, 4, 6–9]. Together, the present findings provide a framework for further study of the molecular evolution of germ line genes in metazoans.

## Methods

### Identification of Germ Line Genes for Analysis

A set of 34 genes with experimental and/or cytological evidence of involvement in PGC-specification under induction mode (*N*=13), inheritance mode (*N*=15), or both modes (*N*=4) as well as two LSI genes *osk* and *pgc* (Table 1), were selected for study in *Drosophila*. CDS from *D. melanogaster* (longest isoform per gene) were used as the reference CDS set for orthology searching as PGC-specification has been well-studied in that organism, its genome has been well annotated (www.flybase.org), and it is arguably the best annotated species in the genus [48]. For study within the genus, five additional species of *Drosophila* within the *melanogaster* group, *D. erecta*, *D. sechellia*, *D. simulans*, *D. yakuba*, were chosen, as well as a relative outgroup species *D. ananassae* (Additional file 1: Figure S1 and Table S1). All six species are closely related taxa and exhibit a range of dN/dS values (Additional file 1: Figure S2) and largely unsaturated dN and dS (Additional file 1: Figure S3), making them suitable for study of molecular evolution [48, 56]. The procedures used for identification of suitable orthologs for study between *Drosophila* and *Caenorhabditis* and *Apis*, as well for among the various species within each genus, are described in detail in Additional file 1: Text file S4.

### Molecular Evolutionary Analysis

The CDS for each gene per genus were aligned by codons using MUSCLE [113] in MEGA [114] set at default parameters with the exception that the gap penalty was set to -1.9, which yielded more effective alignments (than the default of -2.9) across multiple-species. Regions with gaps were removed. It has been proposed that small segments in a gene with poor alignment or greater divergence might influence measures such as dN and dS, and detection of positive selection, and their removal improves such estimates despite loss of some sequence information [115, 116]. Thus, we used a dual approach of filtering using the program GBLOCKS [115] set at default parameters, which accordingly shortened divergent alignments, and inspection of protein alignments by eye, always retaining the start codon [117, 118], to remove residual divergent and putatively misaligned segments. Thus, all alignments and measures of substitution rates herein are considered conservative, and the latter applies specifically to the aligned regions per gene.

Protein sequence divergence per phylogenetic branch was measured using dN and dS under the free ratio model (M1) in codeml of PAML based on an unrooted tree for each genus [55]. Whilst some studies have used the M0 model in PAML to measure dN/dS in taxa including the *melanogaster* group in *Drosophila*, which determines a single dN/dS across all branches in the phylogeny [56], we allowed a separate dN/dS in each branch to include potential species-specific effects on dN/dS (Additional file 1: Figure S2) using the free-ratios model [55]. As noted in the Results and Discussion $\overline{\mathrm{dN}}$/$\overline{\mathrm{dS}}$ (using M1 model) and M0 dN/dS were strongly correlated across genes within each genus (Spearman’s ranked R>0.95, P<2X10^-7^). The value of $\overline{\mathrm{dN}}$/$\overline{\mathrm{dS}}$ was used instead of mean dN/dS across the branches as the latter can be biased towards extremely high values due to rare cases (branches) with extremely low dS and avoids exclusion of a branch (i.e., no dN/dS value) in cases when dN>0 and dS=0 [119]. The phylogeny for *Drosophila* was taken as that provided at FlyBase [109] and was unrooted for PAML. The unrooted *Caenorhabditis* four-species phylogeny was taken from [120] and for *Apis* from [121]. For the latter genus, which is less strongly resolved for (ingroup) positions of *A.dorsata* and *A. cerena*, alternate phylogenies were employed for the ingroup yielding highly similar results. We note that we did not detect orthologs of the germ line gene *par-1* in all six *Drosophila* species, and thus we did not formally include it in the *Drosophila* gene set for study, but a three-species alignment was studied, and *par-1* was examined in *Caenorhabditis* and *Apis*; the results are described in Additional file 1: Text File S5.

Positive selection was assessed using “sites” analysis in PAML across all species per genus [55]. For this we compared M7 versus M8. For those genes exhibiting positive selection using 2X∆lnlikelihood based on the Chi^2^ table, we obtained the BEB posterior probabilities identifying the sites with *P*>0.90.

### Codon Usage Bias

The values of mENC’, which accounts for abundance of rare amino acids and for nucleotide content of the genes under study [61] was conducted using a program from Satapathy et al. [62]. Standard ENC [60], GC3 content at 3^rd^ synonymous codon positions (GC3s) and GC content per CDS was determined in CodonW [122]. For consistency with $\overline{\mathrm{dN}}$/$\overline{\mathrm{dS}}$, all mENC’, ENC and GC values were determined using the aligned sequences per gene excluding gaps for each species.

### Inter-Genus Contrasts

We compared the $\overline{\mathrm{dN}}$/$\overline{\mathrm{dS}}$ values and the mENC’ values of orthologs present in each of the two genera per contrast (cf. [123]). As genes varied in sequence between genera and were aligned separately within each genus, under a conservative approach, we compared the relative ranking of germ line genes within each genus (using Spearman Rank Correlations) to assess any relationships between genera.

### Pleiotropy

For analysis of pleiotropy in *D. melanogaster*, gene expression breadth was determined using the modENCODE transcriptome database as presented at FlyBase.org [109, 124] across 30 tissues and stages of development. These included twelve stages from embryos, six from larvae, six from pupae, and three for adult males and for adult females (shown in Additional file 1: Table S8). Breadth of expression was quantified as the number of developmental stages in which a gene was expressed [83, 106], and was converted into percentages of the 30 stages studied (Additional file 1: Table S8). Analysis was repeated for those using a cutoff of >5 RPKM.

## Additional file

Additional file 1: The file contains the supplementary Tables, Figures and Text which are denoted and Tables S1 to S8, Figures S1, S2,S3 and S4, and Text files S1,S2, S3, S4 and S5. (PDF 509 kb)

Additional file 2: Alignments. (ZIP 213 kb)
