# Supplementary material for: Contrasting patterns of molecular evolution in metazoan germ line genes
Source: BMC Evol Biol. 2019 Feb 11;19:53. doi: 10.1186/s12862-019-1363-x (PMC6371493; doi:10.1186/s12862-019-1363-x)
Supplement: Supplementary file 1 — The file contains the supplementary Tables, Figures and Text which are denoted and Tables S1 to S8, Figures S1, S2,S3 and S4, and Text files S1,S2, S3, S4 and S5. (PDF 509 kb) [file 12862_2019_1363_MOESM1_ESM.pdf]

Additional file 1 containing supplementary materials for

Contrasting patterns of molecular evolution in metazoan germ line genes

Carrie A. Whittle and Cassandra G. Extavour

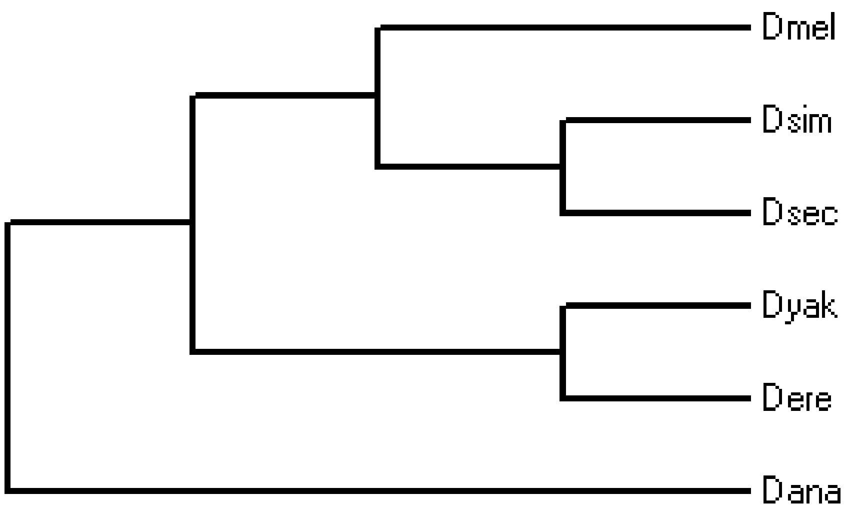

Figure S1. The *Drosophila* phylogeny for six species under study herein. The tree was unrooted for PAML analysis [1] . Each taxon name is abbreviated using the first three letters of the species name. Full names are provided in Table S1. Phylogeny is as provided by FlyBase [2].

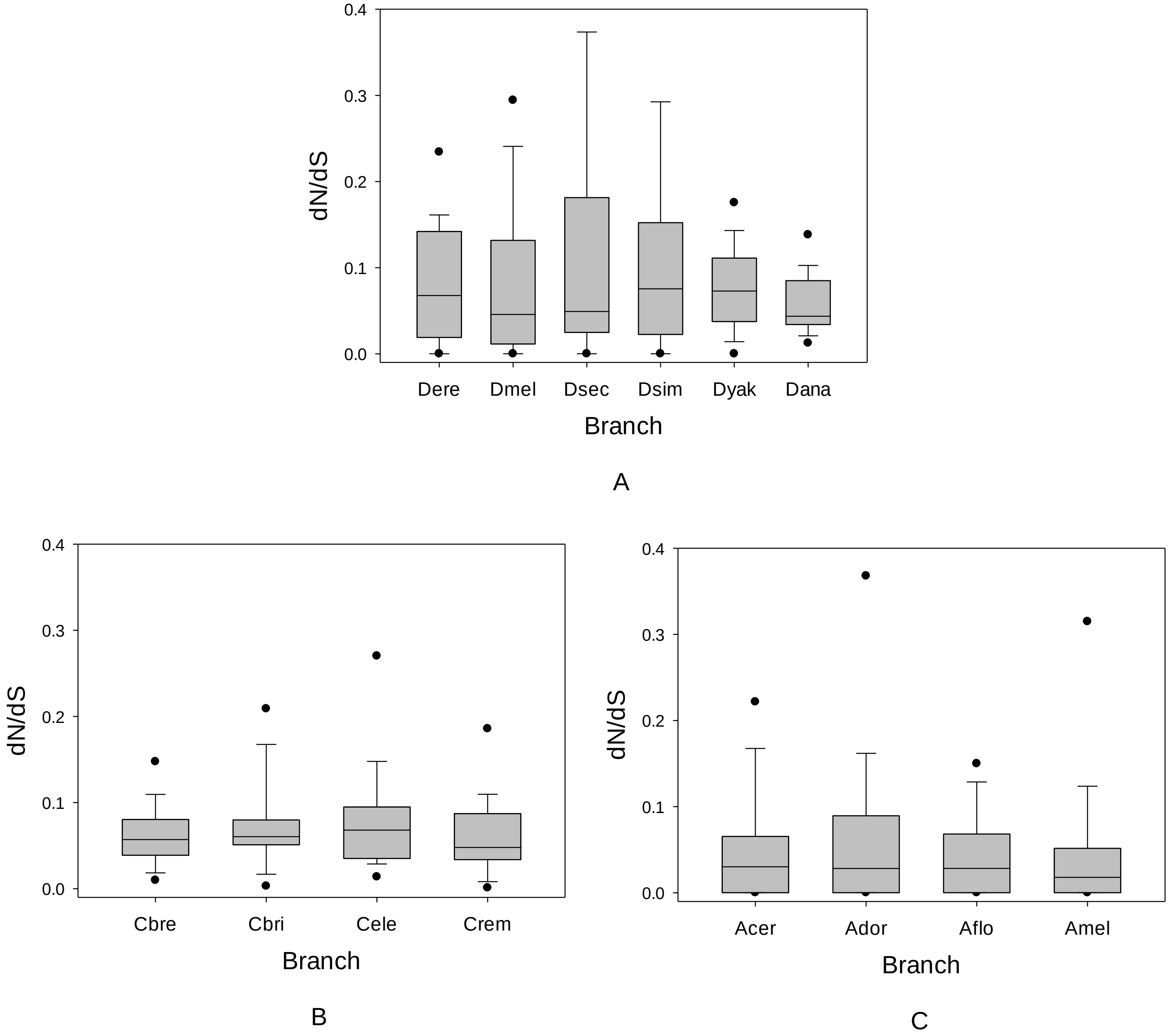

Figure S2. Box plots for dN/dS for each terminal branch per genus. (A) All six *Drosophila* species under study; (B) *Caenorhabditis* species under study; and (C) *Apis* species under study. Each taxon name is abbreviated using the first three letters of the species name.

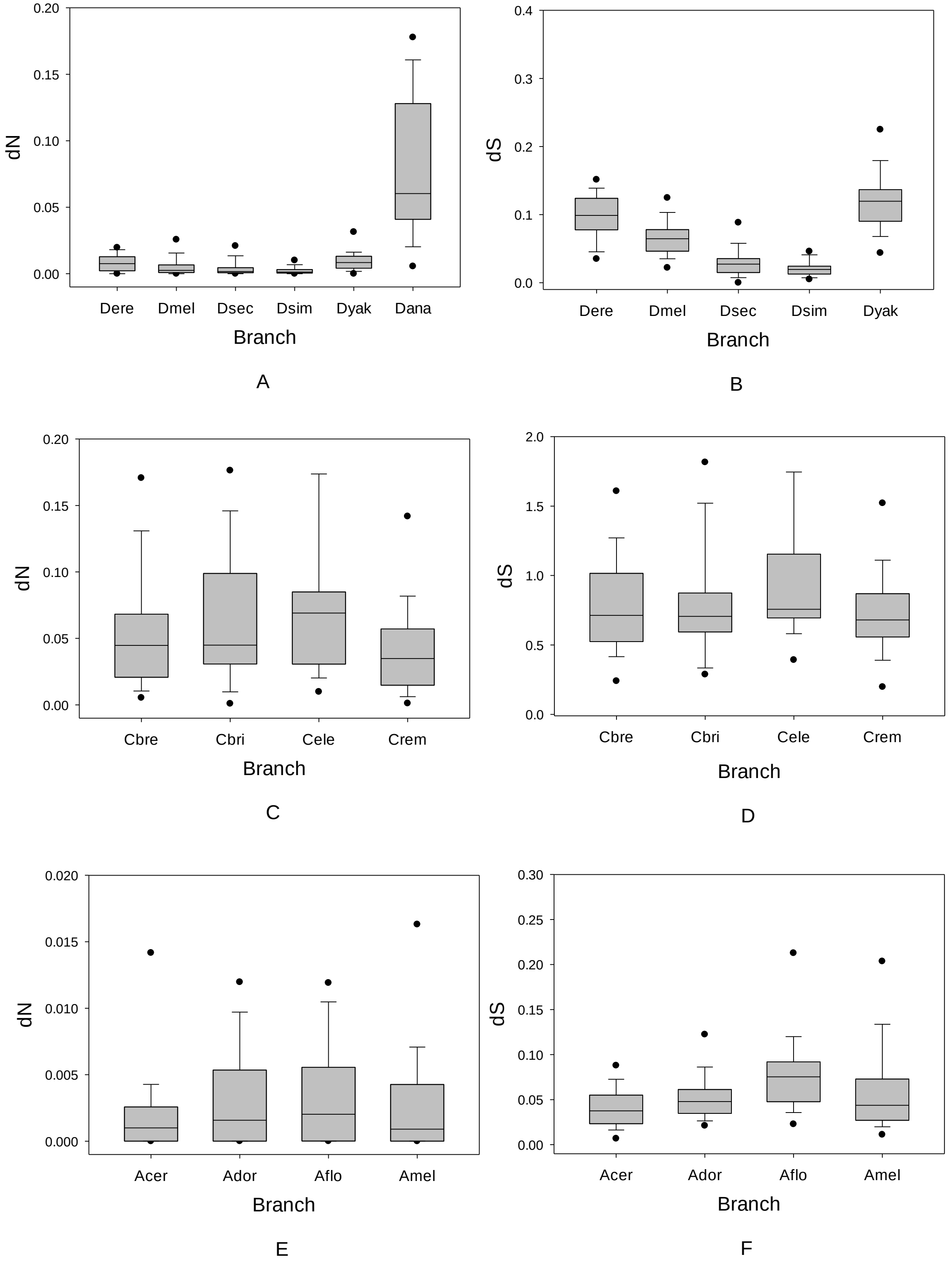

Figure S3. Box plots for dN and dS for all studied genes for each lineage per genus. (A) dN in each *Drosophila* branch; (B) dS in each *Drosophila* branch (C) dN in each *Caenorhabditis* branch; (D) dS in each *Caenorhabditis* branch; (E) dN in each *Apis* branch; (F) dS in each *Apis* branch. Note that for panel B, dS was omitted for *D. ananassae* for visualization purposes, as its values were higher than those of the other species. The median dS for *D. ananassae* was 1.158, and 25th and 75th percentile values were 0.989 and 1.705 respectively, whilst only two values were >2. Each taxon name is abbreviated using the first three letters of the species name.

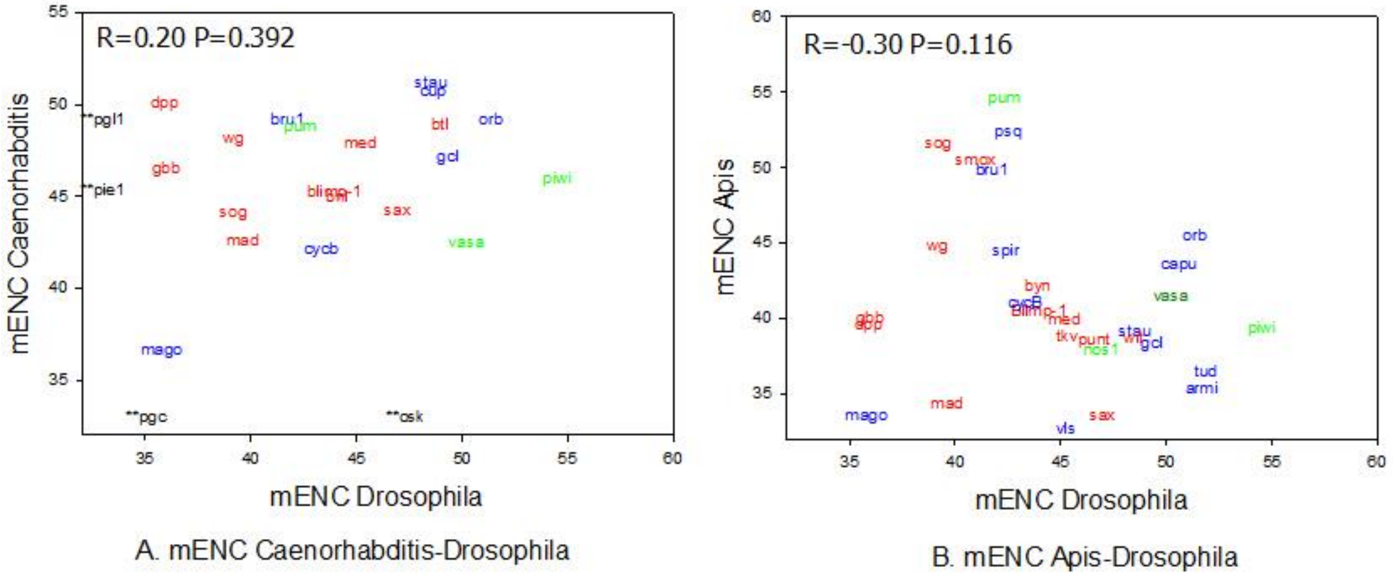

Figure S4. The (A) traditional ENC for *Caenorhabditis* versus *Drosophila* and (B) traditional ENC for *Apis* versus *Drosophila***.** Data points are listed using the gene identifiers. ******Genes that are specific to *Caenorhabditis* or *Drosophila* are shown on the axes. Gene names are those from *Drosophila*.

Table S1. The protein coding sequences (CDS) used in present study to identify germ line genes under study in *Drosophila*, *Caenorhabditis* and *Apis***.** The number of CDS includes all known isoforms.

Taxon	Version	Database[a]	No. CDS
Drosophila melanogaster	release 6.13	Flybase	30,482
D. ananassae	r1.05	Flybase	21,191
D. erecta	r1.05	Flybase	19,592
D. sechellia	r1.3	Flybase	16,471
D. simulans	r2.02	Flybase	24,119
D. yakuba	r1.3	Flybase	16,082
Caenorhabditis elegans	PRJNA13758.WS237	Wormbase	26,189
C. brenneri	PRJNA20035.WS257	Wormbase	30,672
C. briggsae	PRJNA10731.WS257	Wormbase	25,332
C. remanei	PRJNA53967.WS257	Wormbase	31,450
Apis mellifera	Refseq r. 79	Genbank-NCBI	22,460
A. cerena	Refseq r. 79	Genbank-NCBI	19,247
A. dorsata	Refseq r. 79	Genbank-NCBI	18,146
A. florea	Refseq r. 79	Genbank-NCBI	17,664

[a] CDS data are available from FlyBase at http://flybase.org/, for WormBase at http://www.wormbase.org/ and for Genbank-NCBI at https://www.ncbi.nlm.nih.gov/genbank/. Complete and annotated CDS files are available at FlyBase and WormBase for each species under their release number. For *Apis* CDS were downloaded from the Genbank Refseq database using the search criteria: "Species name[organism]". This was followed by up selecting the "Refseq" option for output, and were downloaded using the send to option "coding sequences". The Refseq database contains the non-redundant sequences available for each species, providing a stable reference genome and mRNA. *Apis* sequences were downloaded in December 2016.

Table S2. The 23 *Caenorhabditis* germ line genes under study. Putative *C.elegans* orthologs to the *D. melanogaster* gene list in Table 1 were identified by orthology searches in FlyBase (with *C. elegans* as the taxon of interest). Two germ plasm genes are *Caenorhabditis*-specific (LSI). Only those with orthologs also identified in all three fellow *Caenorhabditis* species (Table S1) using reciprocal BLASTX (with *C. elegans* as the reference) are listed below and were used for analyses.

D. melanogaster	***C. elegans* ortholog**[a]	***C. elegans* Gene ID**
Blimp-1	*blmp-1*	WBGene00003847
bnl	*let-756*	WBGene00002881
bru1	*etr-1*	WBGene00001340
btl	*egl-15*	WBGene00001184
cup	*ifet-1*	WBGene00004132
cycB	*cyb2.1*	WBGene00000866
dpp	*dbl-1*	WBGene00000936
gbb	*tig-2*	WBGene00006570
gcl	*gcl-1*	WBGene00013382
mad	*sma-2*	WBGene00004856
mago	*mag-1*	WBGene00003123
med	*sma-4*	WBGene00004858
NA	*pgl-1*	WBGene00003992
NA	*pie-1*	WBGene00004027
orb	*cpb-3*	WBGene00000772
par-1	*par-1*	WBGene00003916
piwi	*prg-1*	WBGene00004178
punt	*daf-4*	WBGene00000900
sax	*sma-6*	WBGene00004860
sog	*crm-1*	WBGene00007103
stau	*stau-1*	WBGene00018857
vasa	*glh-1*	WBGene00001598
wg	*cwn-1*	WBGene00000857

[a] The *C. elegans* CDS were used as a reference to identify orthologs in the sister species of *Caenorhabditis*. *D. melanogaster* genes (34 in Table 2) that are not listed in the table fell into one of three categories: they did not have high confidence matches in *C. elegans*, the C. *elegans* ortholog also matched another second *D. melanogaster* gene, and/or ortholog matches were not found or poorly aligned across all four *Caenorhabditis* species. *par-1* was added for *Caenorhabditis*, but not studied in all six species in *Drosophila*.

Table S3. The 30 *Apis* genes under study. Putative *A. mellifera* orthologs to the *D. melanogaster* gene list in Table 1 were identified by reciprocal BLASTX. The e-value represents the BLASTX result with *D. melanogaster* CDS as the query against the *A. mellifera* CDS list. As *Apis* has the least well annotated genomes studied, the list of orthologs are putative and comprise the best reciprocal hits to the reference *A. mellifera* and *D. melanogaster* gene list. The *A. mellifera* CDS were used as a reference to identify orthologs in the remaining three sister species of *Apis* using reciprocal BLASTX. Only those genes with putative orthologs in all four *Apis* species are provided below and used for analyses. Functions are those described for the *Apis* CDS from NCBI.

***D. melan.* Gene**	**Gene ID of *Apis mellifera* orthologs in GenBank**	**Putative Function**	**BLASTX e-value**
armi	XM_006571686.2	probable RNA helicase armi	1.00E-130
Blimp-1	XM_006566008.2	probable serine/threonine-protein kinase DDB_G0282963 isoform X1	1.00E-100
bru-1	XM_016911026.1	CUGBP Elav-like family member 2	3E-74
byn	XM_006565966.2	brachyury protein isoform X2	9E-89
capu	XM_016911606.1	protein cappuccino	7E-88
cycB	XM_624245.5	uncharacterized protein LOC551860	5E-65
dpp	XM_006569786.2	protein decapentaplegic	7E-81
gbb	XM_394252.4	protein 60A	1.00E-101
gcl	XM_624700.5	protein germ cell-less isoform X1	1.00E-100
mad	XM_006567242.2	protein mothers against dpp	0
mago	XM_016911522.1	protein mago nashi homolog	4E-76
med	XM_392838.6	mothers against decapentaplegic homolog 4 isoform X1	e-106
nos	XM_016913300.1	protein nanos isoform X1	5E-17
orb	XM_395376.5	cytoplasmic polyadenylation element-binding protein 1 isoform X1	e-120
par-1	XM_006570585.2	serine/threonine-protein kinase MARK2 isoform X2	0
piwi	NM_001165906.1	Aubergine	0
psq	NM_001011602.1	Pipsqueak	2E-95
pum	XM_006565992.2	pumilio homolog 2 isoform X2	0
punt	XM_395928.6	activin receptor type-2A isoform X2	1.00E-150
sax	XM_016917500.1	activin receptor type-1 isoform X1	1.00E-148
smox	XM_006571160.2	mothers against decapentaplegic homolog 3 isoform X1	1.00E-132
sog	XM_393520.6	dorsal-ventral patterning protein Sog isoform X3	0
spir	XM_006570180.2	protein spire isoform X3	8E-91

stau	XM_006564450.2	double-stranded RNA-binding protein Staufen homolog 2 isoform X1	2E-69
tkv	XM_391989.6	bone morphogenetic protein receptor type-1B isoform X1	1.00E-161
tud	XM_016916602.1	LOW QUALITY PROTEIN: maternal protein tudor	8E-53
vasa	XM_006571702.2	ATP-dependent RNA helicase vasa isoform X1	1.00E-149
vls	XM_006565093.2	methylosome protein 50 isoform X1	4E-14
wg	XM_006571239.2	protein Wnt-1	1.00E-169
wit	XM_397334.6	bone morphogenetic protein receptor type-2	1.00E-124

Table S4. $\overline{dN}/\overline{dS}$, mean dN and mean dS and across the phylogeny of six species of *Drosophila* herein for each of the 34 genes under study. Values were measured using codeml in PAML [1]. SE=standard error. Note that additional decimal places of dN and dS than shown were used for calculation of $\overline{dN}/\overline{dS}$.

Gene	$\overline{dN}/\overline{dS}$	Mean dN	SE	Mean dS	SE
**Lineage-specific Inheritance**					
osk	0.1573	0.0437	0.0350	0.2779	0.2290
pgc	0.0933	0.0290	0.0267	0.3108	0.2808
**Inheritance**					
capu	0.1189	0.0301	0.0189	0.2527	0.1908
orb	0.1102	0.0239	0.0162	0.2172	0.1695
stau	0.0885	0.0249	0.0216	0.2818	0.2417
cup	0.0878	0.0305	0.0245	0.3472	0.2967
vls	0.0810	0.0279	0.0203	0.3440	0.3032
armi	0.0773	0.0355	0.0294	0.4596	0.4307
spir	0.0751	0.0133	0.0110	0.1770	0.1309
tud	0.0716	0.0304	0.0242	0.4246	0.3949
cycB	0.0692	0.0158	0.0113	0.2284	0.1911
gcl	0.0392	0.0125	0.0092	0.3189	0.2747
bru1	0.0369	0.0083	0.0072	0.2250	0.1794
psq	0.0297	0.0023	0.0015	0.0789	0.0579
mago	0.0001	0.0000	0.0000	0.2459	0.2003
**Induction**					
bnl	0.1303	0.0328	0.0245	0.2519	0.1829
btl	0.0935	0.0342	0.0287	0.3663	0.3207
wg	0.0833	0.0183	0.0139	0.2199	0.1597
tkv	0.0734	0.0141	0.0091	0.1924	0.1316
byn	0.0575	0.0128	0.0094	0.2230	0.1689
Blimp-1	0.0572	0.0118	0.0070	0.2068	0.1384
dpp	0.0517	0.0123	0.0085	0.2384	0.2028
wit	0.0462	0.0128	0.0099	0.2763	0.2267
med	0.0422	0.0094	0.0085	0.2233	0.2003
sax	0.0402	0.0109	0.0084	0.2712	0.2294
sog	0.0305	0.0080	0.0074	0.2634	0.1913
gbb	0.0303	0.0147	0.0126	0.4865	0.4122
mad	0.0248	0.0053	0.0044	0.2120	0.1752
punt	0.0232	0.0091	0.0070	0.3923	0.3543
smox	0.0135	0.0026	0.0028	0.1933	0.1631
**Inh/Ind**					
nos	0.1145	0.0342	0.0270	0.2984	0.2706
vasa	0.0545	0.0263	0.0139	0.4822	0.4201
piwi	0.0446	0.0214	0.0186	0.4794	0.4394
pum	0.0320	0.0055	0.0045	0.1727	0.1356

Table S5. $\overline{dN}/\overline{dS}$, mean dN and mean dS across the phylogeny of four species of *Caenorhabditis* herein for each of the 23 genes under study. Values were measured using codeml in PAML [1]. SE=standard error. Note that additional decimal places of dN and dS than shown were used for calculation of $\overline{dN}/\overline{dS}$.

***C. elegans* Gene Name**	**DM Name**	**Mean dN/dS**	**Mean dN**	**SE**	**Mean dS**	**SE**
**<u>Lineage-specific Inheritance</u>**						
pie-1	-	0.1619	0.1243	0.0376	0.7681	0.1056
pgl-1	-	0.1553	0.1580	0.0053	1.0170	0.1834
**<u>Inheritance</u>**						
cyb-2	*cyb2*	0.1127	0.0826	0.0498	0.7324	0.2730
cpb-3	*orb*	0.0833	0.0714	0.0127	0.8576	0.1878
gcl-1	*gcl*	0.0719	0.1137	0.0257	1.5809	0.1900
ifet-1	*cup*	0.0570	0.0410	0.0032	0.7192	0.0571
stau-1	*stau*	0.0499	0.0957	0.0083	1.9185	0.4262
par-1	*par-1*	0.0433	0.0209	0.0041	0.4818	0.0576
etr-1	*bru1*	0.0386	0.0334	0.0044	0.8651	0.1563
mag-1	*mago*	0.0081	0.0054	0.0008	0.6591	0.1921
**<u>Induction</u>**						
dbl-1	*dpp*	0.0963	0.0247	0.0092	0.2568	0.0537
let-756	*bnl*	0.0748	0.0732	0.0113	0.9795	0.1237
sma-6	*sax*	0.0675	0.0537	0.0100	0.7961	0.0917
egl-15	*btl*	0.0639	0.0648	0.0189	1.0131	0.1962
blmp-1	*Blimp-1*	0.0575	0.0385	0.0133	0.6698	0.0429
sma-4	*med*	0.0548	0.0383	0.0101	0.6985	0.0689
tig-2	*gbb*	0.0522	0.0349	0.0198	0.6683	0.1161
crm-1	*sog*	0.0395	0.0238	0.0025	0.6027	0.0749
cwn-1	*wg*	0.0264	0.0256	0.0094	0.9688	0.1297
sma-2	*mad*	0.0128	0.0073	0.0058	0.5707	0.0986
**<u>Inh/Ind</u>**						
glh-1	*vasa*	0.0761	0.0419	0.0071	0.5506	0.0809
puf-8	*pum*	0.0753	0.0669	0.0088	0.8878	0.1105
prg-1	*piwi*	0.0642	0.0544	0.0214	0.8474	0.2268

Table S6. $\overline{dN}/\overline{dS}$, mean dN and mean dS across the phylogeny of four species of *Apis* herein for each of the germ line genes under study. Values were measured using codeml in PAML [1]. SE=standard error. Note that additional decimal places of dN and dS than shown were used for calculation of $\overline{dN}/\overline{dS}$.

Gene	$\overline{dN}/\overline{dS}$	Mean dN	SE	Mean dS	SE
**Inheritance**					
armi	0.1011	0.0068	0.0018	0.0673	0.0138
tud	0.0792	0.0054	0.0007	0.0682	0.0102
cycB	0.0774	0.0014	0.0008	0.0176	0.0024
vls	0.0673	0.0038	0.0004	0.0566	0.0170
spir	0.0564	0.0031	0.0012	0.0555	0.0096
bru-1	0.0560	0.0024	0.0028	0.0437	0.0155
stau	0.0395	0.0014	0.0010	0.0347	0.0137
gcl	0.0326	0.0011	0.0008	0.0336	0.0107
capu	0.0238	0.0041	0.0017	0.1699	0.0495
orb	0.0192	0.0006	0.0003	0.0332	0.0065
psq	0.0173	0.0018	0.0013	0.1060	0.0677
par-1	0.0067	0.0002	0.0002	0.0245	0.0041
mago	0.0001	0.0000	0.0000	0.0624	0.0335
**Induction**					
dpp	0.1342	0.0058	0.0044	0.0431	0.0146
sax	0.1163	0.0083	0.0033	0.0714	0.0092
wg	0.0854	0.0042	0.0039	0.0490	0.0082
sog	0.0627	0.0045	0.0024	0.0712	0.0080
wit	0.0462	0.0025	0.0013	0.0542	0.0126
Blimp-1	0.0375	0.0043	0.0017	0.1139	0.0253
gbb	0.0363	0.0015	0.0010	0.0411	0.0133
punt	0.0335	0.0016	0.0006	0.0473	0.0070
byn	0.0324	0.0020	0.0014	0.0624	0.0136
med	0.0133	0.0005	0.0003	0.0352	0.0049
tkv	0.0116	0.0008	0.0006	0.0656	0.0084
smox	0.0080	0.0003	0.0003	0.0314	0.0058
mad	0.0001	0.0000	0.0000	0.0443	0.0136
**Inh/Ind**					
piwi	0.1393	0.0105	0.0030	0.0755	0.0170
vasa	0.1155	0.0093	0.0018	0.0806	0.0144
pum	0.0038	0.0003	0.0003	0.0671	0.0147
nos	<0.0001	0.0000	0.0000	0.0460	0.0087

Table S7. The mean GC content and GC content at 3rd synonymous positions of codons (GC3s) for all genes under study in *Drosophila*, *Caenorhabditis* and *Apis*. SE=standard error.

	Drosophila					***Caenorhabditis***					***Apis***			
**Gene**	**GC**	**SE**	**GC3s**	**SE**	**Gene**	**GC**	**SE**	**GC3s**	**SE**	**Gene**	**GC**	**SE**	**GC3s**	**SE**
armi	0.500	0.011	0.588	0.032	*blmp-1*	0.429	0.020	0.279	0.024	*armi*	0.277	0.001	0.092	0.002
Blimp-1	0.591	0.006	0.708	0.017	*cpb-3*	0.454	0.020	0.373	0.025	*Blimp-1*	0.525	0.005	0.724	0.012
bnl	0.564	0.004	0.699	0.011	*crm-1*	0.449	0.020	0.339	0.038	*bru-1*	0.512	0.003	0.616	0.005
bru-1	0.590	0.004	0.737	0.012	*cwn-1*	0.443	0.021	0.425	0.046	*byn*	0.415	0.002	0.218	0.004
btl	0.541	0.010	0.639	0.028	*cyb-2*	0.487	0.021	0.577	0.034	*capu*	0.528	0.001	0.719	0.007
byn	0.605	0.003	0.687	0.009	*dbl-1*	0.450	0.021	0.395	0.024	*cycB*	0.332	0.002	0.214	0.004
capu	0.564	0.006	0.626	0.017	*egl-15*	0.411	0.022	0.359	0.026	*dpp*	0.368	0.002	0.160	0.006
cup	0.543	0.003	0.663	0.004	*etr-1*	0.474	0.022	0.425	0.042	*gbb*	0.578	0.002	0.734	0.005
cycB	0.561	0.003	0.711	0.008	*gcl-1*	0.384	0.022	0.318	0.012	*gcl*	0.326	0.001	0.174	0.004
dpp	0.623	0.008	0.835	0.021	*glh-1*	0.470	0.020	0.318	0.007	*mad*	0.361	0.001	0.093	0.003
gbb	0.595	0.008	0.822	0.024	*ifet-1*	0.482	0.020	0.388	0.002	*mago*	0.309	0.002	0.126	0.007
gcl	0.534	0.005	0.662	0.008	*let-756*	0.445	0.021	0.338	0.054	*med*	0.415	0.001	0.158	0.003
mad	0.574	0.003	0.772	0.007	*mag-1*	0.502	0.021	0.676	0.032	*nos*	0.429	0.003	0.343	0.004
mago	0.545	0.008	0.789	0.022	*par-1*	0.477	0.020	0.419	0.039	*orb*	0.396	0.001	0.244	0.003
med	0.628	0.002	0.674	0.006	*pgl-1*	0.474	0.020	0.446	0.008	*par-1*	0.455	0.002	0.362	0.006
nos	0.559	0.006	0.656	0.020	*pie-1*	0.480	0.020	0.423	0.026	*piwi*	0.361	0.001	0.158	0.004
orb	0.533	0.005	0.604	0.013	*prg-1*	0.403	0.020	0.513	0.009	*psq*	0.477	0.014	0.464	0.041
osk	0.515	0.004	0.652	0.010	*puf-8*	0.471	0.019	0.387	0.017	*pum*	0.508	0.004	0.410	0.011
pgc	0.540	0.007	0.712	0.015	*sma-2*	0.424	0.019	0.423	0.031	*punt*	0.360	0.001	0.166	0.003
piwi	0.468	0.009	0.513	0.026	*sma-4*	0.446	0.020	0.318	0.065	*sax*	0.321	0.001	0.112	0.003
psq	0.605	.002	0.711	0.006	*sma-6*	0.399	0.020	0.300	0.025	*smox*	0.459	0.001	0.380	0.003
pum	0.618	0.003	0.725	0.010	*stau-1*	0.449	0.015	0.394	0.032	*sog*	0.461	0.005	0.340	0.013
punt	0.541	0.003	0.682	0.010	*tig-2*	0.440	0.012	0.358	0.045	*spir*	0.383	0.001	0.218	0.003
sax	0.531	0.009	0.633	0.024						*stau*	0.384	0.000	0.163	0.001
smox	0.584	0.004	0.751	0.010						*tkv*	0.361	0.001	0.161	0.003
sog	0.596	0.005	0.761	0.013						*tud*	0.293	0.001	0.104	0.003
spir	0.601	0.004	0.765	0.009						*vasa*	0.374	0.003	0.232	0.009
stau	0.560	0.005	0.630	0.012						*vls*	0.303	0.001	0.088	0.005
tkv	0.551	0.006	0.663	0.016						*wg*	0.569	0.001	0.693	0.003
tud	0.486	0.013	0.569	0.038						*wit*	0.322	0.000	0.142	0.003

vasa	0.506	0.013	0.506	0.038								
vls	0.583	0.009	0.687	0.020								
wg	0.577	0.008	0.739	0.019								
wit	0.561	0.004	0.645	0.013								
**Mean**	0.561	0.006	0.683	0.016	0.450	0.020	0.400	0.029	0.405	0.002	0.293	0.006

Table S8. The stages of development wherein expression was quantified in *D. melanogaster* using transcriptome data from www.flybase.org.

Stage of Development
embryo 00-02hr
embryo 02-04hr
embryo 04-06hr
embryo 06-08hr
embryo 08-10hr
embryo 10-12hr
embryo 12-14hr
embryo 14-16hr
embryo 16-18hr
embryo 18-20hr
embryo 20-22hr
embryo 22-24hr
larva L1
larva L2
larva L3 12hr old
larva L3 puffstage 1-2
larva L3 puffstage 3-6
larva L3 puffstage 7-9
white prepupae new
white prepupae 12hr
white prepupae 24hr
pupae 2d postWPP
pupae 3d postWPP
pupae 4d postWPP
adult male 01day
adult male 05day
adult male 30day
adult female 01day
adult female 05day
adult female 30day

Text File S1: Comparison of Drosophila dN/dS to genome-wide values

For our reference and main target taxon *Drosophila*, we assessed the rate of divergence in the PGC-specification gene set to genome-wide values. For the six *Drosophila* species from the *melanogaster* group studied here, a database of dN/dS using the M0 model in PAML [1] has been generated for 8,510 genes with 1:1 single-copy orthologs in all taxa and is available at FlyBase [2, 3]. Using those datasets [3], we determined the average value of dN/dS was 0.0876 (standard error $\pm 9.0\text{X}10^{-4}$) and $\overline{\text{dN}}/\overline{\text{dS}}$ was 0.0837. Using the more recent and updated *Drosophila* database for the *melanogaster* group at flyDIVaS (downloadable version 1, also generated using M0 in PAML) [4], we obtained highly similar results with a genome-wide average value for dN/dS of 0.0864 ± 9.210^{-6} (N=8,656). Based on these values, the *Drosophila* $\overline{\text{dN}}/\overline{\text{dS}}$ results herein for *osk*, *bnl*, *capu*, *nos*, *orb*, *btl* and *pgc* (Table 2: 0.0933 to 0.1573) indicate these genes have diverged at a rate above the genome-wide average, consistent with particularly rapid evolution. Further, the germ line genes when taken as a collective group appear to span values observed in the genome as a whole, and thus do not exhibit strongly conserved or rapidly evolving as compared to the rest of the genome (as a group).

Text File S2:

Positive selection in Drosophila

A prior genome-wide study of the six species from the *Drosophila melanogaster* group has indicated that 878 of 8,150, or 10.7%, of genes examined have been subjected to positive selection [3] based on sites analysis in PAML [1]. This implies that the 34 germ line genes studied herein for *Drosophila* exhibit a lower percentage of genes with positive selection than observed in the genome as a whole; as we found relatively few genes (only 2 of 34 studied, or 6%) exhibited positive selection at codon sites (Table 5). However, this may be partly due to our conservative method of generating alignments: we aimed to improve divergence estimates by using GBLOCKS on our MEGA alignments [5] to remove any divergent segments that may have contained misaligned regions [6], and further aligned sequences by eye to remove residual ambiguous and divergent segments [7, 8]. We chose this conservative approach as prior data has demonstrated that ambiguous alignments can result in inflated estimates of positive selection at specific sites (e.g., ≥48% false positives), including those reported for the *Drosophila* genome-wide analyses [3, 9, 10]. Excluding the estimated 48% of false positives suggested to have occurred in the whole-genome *Drosophila* dataset [3] we are left with 52% (estimated true positives) of the 10.7% of genes in the genome that show signs of positive selection. This, level of positive selection (5.6%) is highly similar to that found in our gene line gene set in this taxon (6%), suggesting that the germ line genes studied here exhibit levels of positive selection that are typical for genes in the *Drosophila* genome as a whole. Nonetheless, whist our approach was highly conservative, we do not fully exclude the possibility that some genes in Table 5 might still have occasionally exhibited positive selection due to alignment ambiguity at specific sites [10].

Positive selection in Apis

It is worthwhile mentioning that among the three genera under study herein, sites analysis in PAML suggested positive selection was uncommon for *Drosophila* and *Caenorhabditis* germ line genes (found for two and three genes respectively, Table 5) and was most common in *Apis* (nine of the 30 genes; M7 versus M8 2XΔlnL >5.99, P<0.05, Table 5). *dpp* was near statistical significance (2XΔlnLiklihood=5.56, P=0.062) in *Apis*, with four codon sites showing signs of positive selection by BEB posterior possibilities (P>0.90)). The genes exhibiting positive selection in *Apis* span all categories of germ line specification studied (categories 2-4 as there

were no lineage-specific genes in *Apis*, Table 5). Nonetheless, while positive selection appears most common in this genus, under a conservative interpretation, we note that *Apis* contains the least well-annotated genomes of the three genera studied here. Thus, we cannot fully exclude that the issues inherent to positive selection tests, which have been reported to be very site-sensitive and to have been consistently overestimated by up to an order of magnitude in the literature (as an example, reportedly overestimated in chimpanzees vs. humans) when based on genomes containing sequencing errors, incomplete annotation, imprecise ortholog matching, and ambiguous alignment segments [9], might partly contribute towards these findings. As an example, the alignment of *dpp* using BLASTp [11] yields an alignment nearly identical to that obtained using our alignment approach (see Methods), but that differs slightly (albeit is equally divergent) in positions 80-87, the precise segment of the protein in which we noted positive selection (Table 5). Thus even these conservative alignments are not perfectly unambiguous. Nevertheless, the elevated propensity for positive selection in the germ line genes in *Apis* suggests a striking difference from *Drosophila* and *Caenorhabditis*, and thus warrants further study as more data become available.

Text File S3: Expression breadth and gene evolution

It is notable that *nos*, the fastest evolving Inh/Ind gene, and *bnl* and *btl*, the fastest evolving Induction genes in *Drosophila* ($\overline{dN}/\overline{dS}$ = 0.1145, 0.1303 and 0.0935 respectively), each had low expression breadth using the >5 RPKM criterion in all 30 developmental stages (20 to 26.7%, Fig. 2BD). In contrast, genes that evolved exceptionally slowly, with $\overline{dN}/\overline{dS} \leq 0.0232$ ((e.g. *mago*, *punt*, and *smox*) were expressed in all 30 tissues using the criterion of >0 and >5RPKM (Fig. 2A-D). These trends in germ line specification genes are consistent with greater conservation of widely expressed genes, as has been often reported in animals [12-14]. Nonetheless, this pattern was not observed universally, as *stau* had 100% expression breadth but relatively elevated $\overline{dN}/\overline{dS}$ (0.0885) within the Induction group (ranked 3rd), while the Inh/Ind genes *vasa* and *piwi* displayed relatively low $\overline{dN}/\overline{dS}$ despite having low expression breadth (≤20%) using the >5RPKM criterion (Fig. 2A-D). Collectively, these results suggest that the level of pleiotropy likely significantly contributes to molecular evolution of most germ line genes. Further, low pleiotropy may contribute to the fast evolution of LSI genes (Table 2, Fig. 2B), where it may promote reduced interference from functions in other non-PGC tissues and thus facilitate positive selection [14] in these genes.

While *nos* was the fastest evolving Inh/Ind gene in *Drosophila* ($\overline{dN}/\overline{dS}$ =0.1145, mENC'=50.5 (±0.40)), and had comparatively low pleiotropy (Fig. 1B), the putative orthologous gene identified in *Apis* was surprisingly extremely conserved ($\overline{dN}/\overline{dS}$ =0.0001, mENC'= 38.9 (±0.1) (Fig. 2B, Table 4). We note also that the inter-genus orthologs were very divergent in protein sequence for *nos* between these genera, as observed previously for this gene across animal models [15]. As further developmental expression data becomes available for *Apis*, it will be worthwhile to assess whether high expression breadth is observed for the *nos* gene in that taxon (including embryonic expression [16]), putatively contributing to the much different evolution of this gene in these two genera. In addition, it is worth consideration of whether another putative *nos* ortholog can be identified in *Apis*.

Text File S4: Additional methods

To identify orthologs in all six *Drosophila* species, we compared the 34 CDS of *D. melanogaster* (using longest isoform per gene in the reference species, with rare exception the second longest) to the complete CDS list of each of the five remaining species (including all known isoforms, Table S1) using legacy BLASTX [11] as is employed in FlyBase [2], and for consistency was also used in standalone BLASTX (v. 2.2.18) searches; all cited e-values were obtained from that approach. The modified version BLAST+, which contains the same core programs (e.g., BLASTX), yielded the same interspecies matches per gene (with typically slightly lower e-values, data not shown). The best hit with the lowest e-value and $e<10^{-6}$ was taken as the match. Reciprocal searches were conducted using the best hit for each CDS back to the complete *D. melanogaster* CDS list. Those CDS that were best hits in reciprocal BLASTX searches were used for analysis. All gene identifiers of orthologs across species concurred with those predicted in the www.flybase.org orthology search tool. In some cases, a well-established germ line gene was part of a gene family in *D. melanogaster*: in those cases, we studied the single gene (or copy) most strongly linked to PGC-specification based on the literature, and its best match ortholog in compared taxa (Table 1). Thus, our analysis is of one-to-one best match orthologs of germ line genes [17], and excludes paralogs with presumably relatively weak or absent germ line roles, as such paralogous genes have often undergone changes of substrate or ligand specificity [17, 18].

For *Caenorhabditis* and *Apis*, we examined four model species wherein the genomes were sufficiently complete to allow identification of orthologs (containing the full CDS) for the genes under study (Table S1). The CDS list for the well-studied system *C. elegans* was used as the reference species for *Caenorhabditis*. In the nematodes, we identified orthologs to the gene list from *Drosophila* (Table 1) using the Orthology Search tool available at FlyBase (www.flybase.org) with the *D. melanogaster* FlyBase gene identifier as input, and *C. elegans* as the target taxon of interest. Using the resultant gene list in *C. elegans*, plus three additional nematode-specific genes involved in germ plasm (*pgl-1*, *pie1*, and *meg-1*) we searched for orthologs in the remaining three *Caenorhabditis* species under study, *C. brenneri*, *C. briggsae*, and *C. remanei*, using reciprocal BLASTX searches as described above. For *Apis*, where orthology to *D. melanogaster* genes has been less well studied and thus unavailable in orthology search tools, we conducted reciprocal BLASTX of the 34 *D. melanogaster* CDS (plus *par-1*) to

the complete CDS list for the most well studied model *A. mellifera*. Using the gene list from *A. mellifera*, we then searched for orthologs in the three additional *Apis* species, *A. cerena*, *A. dorsata*, and *A. florea*, using reciprocal BLASTX (S1 Table). The gene *par-1* was included for *Caenorhabditis* and *Apis*, but was not included the formal gene list for *Drosophila* as only three alignable orthologs (to the longest *D. melanogaster* isoform) were found among the six fly species. Results for *par-1* are provided in Supporting Text File 5. The unique gene identifiers for all genes studied per genus have been provided in Table 1 and Tables S2-S3. The final gene list for *Drosophila*, *Caenorhabditis* and *Apis* contained 34, 23 and 30 genes respectively.

For all genera, ortholog matches were further confirmed using alignments (see below). Only genes that had quality matches from ortholog searches (criteria described above) in all species under study per genus and could be confidently aligned were further examined. When two or more CDS, including isoforms for a single gene, had an identical e-value in a BLASTX search, we chose the one with the highest bit score as the best match. Using different isoforms may alter dN/dS or CUB marginally for some genes, but for consistency we always chose the isoform with the best reciprocal BLAST hit to the reference species. Occasional CDS with ambiguous sites were processed in ORF-predictor [19], which sometimes yielded a considerably shortened but highly reliable ORF (e.g., *tud* in *Drosophila*) and alignment for a gene.

Finally, we note that the *D. melanogaster* genes (34 in Table 2) that are not listed as having orthologs in *Caenorhabditis* (Table 3, Table S2) or in *Apis* (Table 4, Table S3) fell into one of three categories: 1) they did not have high confidence matches in *C. elegans* or *A. mellifera* using criteria defined above; 2) the C. *elegans* or *A. mellifera* ortholog also matched another second *D. melanogaster* gene; and/or 3) the ortholog matches were not found or poorly aligned across all four *Caenorhabditis* species or among all four *Apis* species. We chose to employ a conservative approach to ortholog identification in the study and thus any gene that fell into any one of these categories was excluded from analysis in the respective genus. Thus, we do not exclude that some of these genes excluded have orthologs, but did fall not within these criteria.

Text File S5: Results for par-1

It is noteworthy that for the gene *par-1*, which is involved in cell polarity and in stabilization of Osk in *D. melanogaster* and PIE-1 in *Caenorhabditis* [20-23] we could identify unambiguous orthologs for the longest isoform in only three of the *Drosophila* species studied here (*D melanogaster*, *D. erecta, D. simulans*); we thus did not include it in Table 2. Nonetheless using CDS for those three species, we found a $\overline{dN}/\overline{dS}$ of 0.0672, which would place it below the 10th place of the 13 Inheritance genes in Table 2. For *Caenorhabditis*, where we found *par-1* orthologs in all four species studied, we observed *par-1* had also had a relatively low $\overline{dN}/\overline{dS}$ within that genus (0.0433), well below the median for Inheritance genes (0.0751). However, it did exhibit signs of positive selection (2XΔLnLikelihood=22.5, P<0.05), with three sites identified by BEB analysis (P>0.95, and six sites with P>0.90, Table 5), suggesting adaptive evolution of this gene in nematodes, in *Apis*, the *par-1* gene evolved remarkably slowly with a value of 0.0067 (2nd slowest behind *mago*). Speculatively, the *par-1* protein might have been subjected to adaptive evolution in *Drosophila*, possibly in response to rapid changes observed in its phosphorylation target protein Osk [22, 24, 25], which had the highest $\overline{dN}/\overline{dS}$ (Table 2).

References

1. Yang Z: **PAML 4: phylogenetic analysis by maximum likelihood**. *Molecular Biology and Evolution* 2007, **24**(8):1586-1591.

2. Gramates LS, Marygold SJ, Santos GD, Urbano JM, Antonazzo G, Matthews BB, Rey AJ, Tabone CJ, Crosby MA, Emmert DB *et al*: **FlyBase at 25: looking to the future**. *Nucleic Acids Res* 2016.

3. Clark AG, Eisen MB, Smith DR, Bergman CM, Oliver B, Markow TA, Kaufman TC, Kellis M, Gelbart W, Iyer VN *et al*: **Evolution of genes and genomes on the *Drosophila* phylogeny**. *Nature* 2007, **450**(7167):203-218.

4. Stanley CE, Jr., Kulathinal RJ: **flyDIVaS: A Comparative Genomics Resource for Drosophila Divergence and Selection**. *G3 (Bethesda)* 2016, **6**(8):2355-2363.

5. Kumar S, Stecher G, Tamura K: **MEGA7: Molecular Evolutionary Genetics Analysis Version 7.0 for Bigger Datasets**. *Mol Biol Evol* 2016, **33**(7):1870-1874.

6. Talavera G, Castresana J: **Improvement of phylogenies after removing divergent and ambiguously aligned blocks from protein sequence alignments.** *Systematic Biology* 2007, **56**(4):564-577.

7. Carr M, Richter DJ, Fozouni P, Smith TJ, Jeuck A, Leadbeater BSC, Nitsche F: **A six-gene phylogeny provides new insights into choanoflagellate evolution**. *Mol Phylogenet Evol* 2017, **107**:166-178.

8. Heidel AJ, Kiefer C, Coupland G, Rose LE: **Pinpointing genes underlying annual/perennial transitions with comparative genomics**. *BMC Genomics* 2016, **17**(1):921.

9. Schneider A, Souvorov A, Sabath N, Landan G, Gonnet GH, Graur D: **Estimates of positive Darwinian selection are inflated by errors in sequencing, annotation, and alignment**. *Genome biology and evolution* 2009, **1**:114-118.

10. Markova-Raina P, Petrov D: **High sensitivity to aligner and high rate of false positives in the estimates of positive selection in the 12 Drosophila genomes**. *Genome Res* 2011, **21**(6):863-874.

11. Altschul SF, Gish W, Miller W, Myers EW, Lipman DJ: **Basic local alignment search tool.** *Journal Of Molecular Biology* 1990, **215**(3):403-410.

12. Duret L, Mouchiroud D: **Determinants of substitution rates in mammalian genes: expression pattern affects selection intensity but not mutation rate**. *Mol Biol Evol* 2000, **17**(1):68-74.

13. Subramanian S, Kumar S: **Gene expression intensity shapes evolutionary rates of the proteins encoded by the vertebrate genome**. *Genetics* 2004, **168**(1):373-381.

14. Mank JE, Ellegren H: **Are sex-biased genes more dispensable?** *Biol Lett* 2009, **5**(3):409-412.

15. Subramaniam K, Seydoux G: ***nos-1* and *nos-2*, two genes related to *Drosophila nanos*, regulate primordial germ cell development and survival in *Caenorgabditis elegans***. *Development* 1999(126):4861-1871.

16. Dearden PK: **Germ cell development in the Honeybee (*Apis mellifera*); *vasa* and *nanos* expression**. *BMC Developmental Biology* 2006, **6**:6.

17. Hulsen T, Huynen MA, de Vlieg J, Groenen PM: **Benchmarking ortholog identification methods using functional genomics data**. *Genome Biol* 2006, **7**(4):R31.

18. Li WH, Yang J, Gu X: **Expression divergence between duplicate genes**. *Trends Genet* 2005, **21**(11):602-607.

19. Min XJ, Butler G, Storms R, Tsang A: **OrfPredictor: predicting protein-coding regions in EST-derived sequences.** *Nucleic Acids Res* 2005, **33**(Web Server issue):W677-680.

20. Shulman JM, Benton R, St. Johnston D: **The Drosophila Homolog of *C. elegans* PAR-1 Organizes the Oocyte Cytoskeleton and Directs *oskar* mRNA Localization to the Posterior Pole**. *Cell* 2000(101):377-388.

21. Reese KJ, Dunn MA, Waddle JA, Seydoux G: **Asymmetric segregation of PIE-1 in C. elegans is mediated by two complementary mechanisms that act through separate PIE-1 protein domains**. *Mol Cell* 2000, **6**(2):445-455.

22. Riechmann V, Gutierrez GJ, Filardo P, Nebreda AR, Ephrussi A: **Par-1 regulates stability of the posterior determinant Oskar by phosphorylation.** *Nature cell biology* 2002, **4**(5):337-342.

23. Morais-de-Sa E, Vega-Rioja A, Trovisco V, St Johnston D: **Oskar is targeted for degradation by the sequential action of Par-1, GSK-3, and the SCF(-)Slimb ubiquitin ligase**. *Dev Cell* 2013, **26**(3):303-314.

24. Benton R, Palacios IM, St Johnston D: **Drosophila 14-3-3/PAR-5 is an essential mediator of PAR-1 function in axis formation**. *Dev Cell* 2002, **3**(5):659-671.

25. Ephrussi A, Lehmann R: **Induction of germ cell formation by *oskar***. *Nature* 1992, **358**(6385):387-392.
